# Overexpression of human alpha-Synuclein leads to dysregulated microbiome/metabolites with ageing in a rat model of Parkinson disease

**DOI:** 10.1186/s13024-023-00628-1

**Published:** 2023-07-04

**Authors:** Yogesh Singh, Christoph Trautwein, Joan Romani, Madhuri S. Salker, Peter H. Neckel, Isabel Fraccaroli, Mahkameh Abeditashi, Nils Woerner, Jakob Admard, Achal Dhariwal, Morten K. D. Dueholm, Karl-Herbert Schäfer, Florian Lang, Daniel E. Otzen, Hilal A. Lashuel, Olaf Riess, Nicolas Casadei

**Affiliations:** 1 Institute of Medical Genetics and Applied Genomics, University of Tübingen, Calwerstaße 7, 72076 Tübingen, Germany; 2 NGS Competence Centre Tübingen (NCCT), University of Tübingen, Calwerstaße 7, 72076 Tübingen, Germany; 3 Research Institute of Women’s Health, University of Tübingen, Calwerstaße 7/6, 72076 Tübingen, Germany; 4 Werner Siemens Imaging Centre (WSIC), Department of Preclinical Imaging and Radiopharmacy, University of Tübingen, Röntgenweg 13, 72076 Tübingen, Germany; 5 School of Life Sciences, Institute of Bioengineering, Laboratory of Molecular and Chemical Biology of Neurodegeneration, École Polytechnique Fédérale de Lausanne (EPFL), SV LMNN Station 19, 1015 CH Lausanne, Switzerland; 6 Institute of Clinical Anatomy and Cell Analysis, University of Tübingen, Österbergstraße 3, 72074 Tübingen, Germany; 7 Institute of Oral Biology, University of Oslo, Sognsvannsveien 10, 0316 Oslo, Norway; 8 Department of Chemistry and Bioscience, Aalborg University, Fredrik Bajers Vej 7H, 9220 Aalborg, Denmark; 9 Enteric Nervous System Working Group, University of Applied Sciences Kaiserslautern, Zweibrücken Campus, Amerikastrasse 1, 66482 Zweibrücken, Germany; 10 Institute of Vegetative Physiology, University of Tübingen, Wilhelmstaße 56, 72074 Tübingen, Germany; 11 Interdisciplinary Naonscience Center (iNANO), Aarhus University, Gustav Wieds Vej 14, 8000 Aarhus C, Denmark

**Keywords:** Gut microbiome, PD, Intestinal inflammation, α-Synuclein, Antibiotics

## Abstract

**Background:**

Braak’s hypothesis states that sporadic Parkinson’s disease (PD) follows a specific progression of pathology from the peripheral to the central nervous system, and this progression can be monitored by detecting the accumulation of alpha-Synuclein (α-Syn) protein. Consequently, there is growing interest in understanding how the gut (commensal) microbiome can regulate α-Syn accumulation, as this could potentially lead to PD.

**Methods:**

We used 16S rRNA and shotgun sequencing to characterise microbial diversity. ^1^H-NMR was employed to understand the metabolite production and intestinal inflammation estimated using ELISA and RNA-sequencing from feces and the intestinal epithelial layer respectively. The Na^+^ channel current and gut permeability were measured using an Ussing chamber. Immunohistochemistry and immunofluorescence imaging were applied to detect the α-Syn protein. LC-MS/MS was used for characterization of proteins from metabolite treated neuronal cells. Finally, Metascape and Ingenuity Pathway Analysis (IPA) bioinformatics tools were used for identification of dysregulated pathways.

**Results:**

We studied a transgenic (TG) rat model overexpressing the human SNCA gene and found that a progressive gut microbial composition alteration characterized by the reduction of Firmicutes to Bacteroidetes ratio could be detected in the young TG rats. Interestingly, this ratio then increased with ageing. The dynamics of *Lactobacillus* and *Alistipes* were monitored and reduced *Lactobacillus* and increased *Alistipes* abundance was discerned in ageing TG rats. Additionally, the SNCA gene overexpression resulted in gut α-Syn protein expression and increased with advanced age. Further, older TG animals had increased intestinal inflammation, decreased Na^+^ current and a robust alteration in metabolite production characterized by the increase of succinate levels in feces and serum. Manipulation of the gut bacteria by short-term antibiotic cocktail treatment revealed a complete loss of short-chain fatty acids and a reduction in succinate levels. Although antibiotic cocktail treatment did not change α-Syn expression in the enteric nervous system of the colon, however, reduced α-Syn expression was detected in the olfactory bulbs (forebrain) of the TG rats.

**Conclusion:**

Our data emphasize that the gut microbiome dysbiosis synchronous with ageing leads to a specific alteration of gut metabolites and can be modulated by antibiotics which may affect PD pathology.

**Supplementary Information:**

The online version contains supplementary material available at 10.1186/s13024-023-00628-1.

## Background

The traditional hallmark of Parkinson’s disease (PD) is the presence of Lewy bodies (LBs), Lewy neurites (LNs) and the loss of dopaminergic (DA) neurons within the *substantia nigra pars compacta* (*SNpc*) [1–4]. LBs and LNs are structures resulting from the misfolding and aggregation of the alpha-Synuclein (α-Syn) protein [4]. The exact mechanisms leading to the aggregation of α-Syn and contributing to its toxicity are still unclear. Notably, the dopaminergic neurons of the *SNpc* appear particularly vulnerable to the effects of α-Syn aggregates, which correlates to the clinical phenotype [4]. Surprisingly, new data have revealed that LBs and LNs are also found in the periphery including in the nerves of the gastrointestinal tract (GIT) [5].

Although PD is classically classified as a disease of the central nervous system, numerous observations suggest that PD is not a single-trait disease [6] and that in a specific form of PD (non-motor symptoms and peripheral neuroinflammation) originates from peripheral tissues such as the gut epithelial cells called enteroendocrine cells (EECs), neurons of the GIT and olfactory system [2, 7–10]. A recent seminal study also reveals that disruption in the gut function, initiation of inflammation, α-Syn histopathology and motor defects in older mice are accelerated when injected with α-Syn fibrils but not with α-Syn monomers [11]. Further, it is also suggested that amyloidogenic α-Syn seeds may transfer from neuron to neuron as a result of retrograde transport causing accumulation of α-Syn in the bowel [2, 12–15]. In the early stages of the disease, PD patients frequently exhibit non-motor symptoms such as olfactory malfunction, constipation and depression [16]. GIT dysfunction (in particular constipation) is observed in approximately 80% of PD patients (sporadic and familial forms) before the occurrence of motor symptoms [16, 17]. However, it remains uncertain where the initial α-Syn aggregates originate. A recent study indicated two subtypes of PD could exist namely (1) brain-to-gut—prodromal phenotype in rapid eye movement (REM) sleep behaviour disorder patients and (2) gut-to-brain—enteric or peripheral autonomic nervous system (Braak’s theory) with respect to the spread of α-Syn pathology in patients, thus, could clarify the issue with different potentially conflicting earlier reports in the PD field [18].

Several studies have highlighted an association of gut microbiome dysbiosis in PD patients compared with healthy controls [1, 19, 20]. Whilst, individual bacterial composition varied from different PD-cohorts, only a few bacterial genera appeared to be consistent among the different studies (e.g. *Bifidobacterium, Akkermansia and Lactobacillus*) [21, 22]. Some were conflicting, in particular the *Lactobacillus* genus [23] with most findings with large-scale data sets different geographical locations suggesting a higher abundance [19, 24–29] whilst a few others highlight a lower abundance of *Lactobacillus* in fecal and blood microbiomes with a limited number of PD patients [20, 30, 31] or no change in naïve de novo PD patients from the Netherlands and Finland [32]. However, the major limitation of these studies is that the (PD patients) microbiome was quantified when the disease was already established. Further, it is also not known whether these changes in the gut microbiome described, play a causative role in the pathogenesis or are merely a consequence of disease [5]. Studying the microbiome before disease onset in humans is not possible due to the unavailability of any known pre-clinical markers. Thus, rodent models are critical in understanding the physiological role of the gut microbiome on disease development [33].

In this context, the presence of synucleinopathy in the autonomic nervous system, accumulation of α-Syn in the bowel as well as altered intestinal microbiota correlated to motor symptoms point to a potential origin of synucleinopathy in the gut of PD patients [34]. Gut bacteria can control the differentiation and function of immune cells in the intestine, periphery and brain [35–37]. A murine model that overexpressed the human α-Syn under the thy1 promoter (Thy1-α-Syn) suggested that the gut microbiome transplants from PD patients into these mice induced parkinsonian-like motor dysfunction and that the microbiome was necessary to induce α-Syn pathology, neuroinflammation and motor defects [33]. Furthermore, using germ-free (GF) environmental conditions, this study showed that the addition of short-chain fatty acids (SCFAs) to GF Thy1-α-Syn mice (which do not have SCFAs due to GF status) is sufficient to recapitulate a colonized phenotype of the Thy1-α-Syn model [33]. These SCFAs, especially butyrate, protect against intestinal hyperpermeability and decrease inflammation through the modulation of microglial activation [38]. Additionally, SCFA-producing bacteria are less abundant in PD patients [39]. However, the exact role of SCFAs in neurodegeneration requires further investigation due to conflicting results in murine and PD patients. Interestingly, antibiotic treatment in Thy1-α-Syn mice improved the fine and gross motor function as well as gut motility defects highlighting that microbial signals modulate the α-Syn-dependent pathology [33], thus providing a pivotal link between the gut microbiome and PD pathology. This finding suggests a potential influence of genetics on gut-microbiome interactions and synucleinopathies [33]. However, how a specific gut bacterial genera/species interacts with its host genetics with progressive ageing leading to PD-like disease progression remains unknown.

Therefore, we hypothesized that α-Syn overexpression could be involved in modulating the gut microbiome composition and metabolite production which results in the induction of α-Syn misfolding and aggregation in enteric neurons. Accordingly, studying the microbiome dynamics, GIT physiology and function could lead to vital new insights into PD pathology. Given the broad pathophysiological effects of PD, we have used a previously described rat model overexpressing full-length non-mutated human α-Syn which includes the entire *SNCA* sequence with upstream regulatory promoter sequences and a flanking downstream region in a BAC construct [40]. Further, the rat bacterial artificial chromosome (BAC)-human SNCA transgenic PD (referred to as TG) model was shown to present a strong overexpression of α-Syn in the brain and to also reproduce the formation of a pathological form of α-Syn. These TG rats develop early changes in novelty-seeking, avoidance, locomotor decline and an early smell deficit before the progressive motor deficit [40]. The observed pathological changes were linked with severe loss of structural dopaminergic integrity [40, 41]. Moreover, the dual-hit hypothesis suggested that pathological α-Syn aggregation starts in the olfactory bulbs and enteric nervous system (ENS) and spreads to the brain [42]. These TG rats also showed that when α-Syn fibrils were injected in the duodenum, propagation of α-Syn pathology occurs bidirectionally and trans-synaptic parasympathetic and sympathetic pathways via the vagus nerve, i.e., duodenum-to-brainstem-to-stomach [43]. This TG rat model holds a close resemblance to the human PD disease progression, therefore, this model appeared to be ideal for testing the hypothesis that α-Syn expression regulates the gut bacterial abundance and pathophysiology of synucleinopathies.

## Methods

### Aim, design and setting of the study

We aim to address our research question – how does microbial diversity affect PD pathology with ageing at the molecular level and can early intervention in the microbiome modulate the disease course? Herein, we have studied longitudinally the gut microbiome dynamics with ageing and report that with ageing, overexpression of human α-Syn changes the gut microbiota composition, metabolites and inflammation. Together, the humanized rat-model suggests that alterations in α-Syn overexpression are more likely responsible for the differences in gut microbiota and the gut microbiome could potentially shape the progression of PD pathogenesis. Furthermore, the bacterial metabolite succinate is identified as responsible for the control of neuro-inflammation in neuronal cells.

### Characteristics of participants or description of materials (animals), genotyping and breeding for microbiome study

To understand if the expression of full–length α-Syn may induce changes in the gastrointestinal microbiome, we used a transgenic rat model of PD overexpressing the human SNCA gene under the human promoter including all the introns [40]. In contrast to other animal models using artificial promoters to drive the transgene expression in specific cell types, we found in our model overexpression of the human protein with a spatial distribution similar to endogenous human and rat expression in the brain [40]. Thus, these TG rats could be an ideal model to understand the gut-brain function.

The BAC-hSNCA TG rats have been described in detail earlier and these animals were maintained under outbred conditions on a Sprague Dawley (SD) background and genotyped as previously mentioned [40]. In brief, to distinguish between homozygous and heterozygous animals, the relative number of DNA copies was estimated by quantitative real-time PCR (q-RT-PCR) on a LightCycler 2.0 (Roche) using a LightCycler FastStart DNA MasterPLUS SYBR Green I kit (Roche) from genomic DNA of rat ear biopsies. Reactions were performed in 10 µL of mixture containing 10 pmol of each primer, 40 ng DNA and 1 × SYBR Green Mix (Roche). Quantitative PCR was carried out in triplicates and normalized to a reference gene (β-actin; β-actin-F: 5’-AGCCATGTACGTAGCCATCCA-3’; β-actin-R: 5’-TCTCCGGAGTCCATCACAATG-3’). Primer sequences to detect the copy number of the α-Syn transgene were located in the promoter sequence (SynProm-F: 5’-CCGCTCGAGCGGTAGGACCGCTTGTTTTAGAC-3’; LC-SynProm-R: 5’-CCTCTTTCCACGCCACTATC-3’). The amplification conditions were as follows: 10 min at 95^ο^C; 45 cycles of 20 seconds (s) at 95^ο^C, 20 s at 58^ο^C, 20 s at 72^ο^C; melting curve: 10 s at 95^ο^C, 20 s at 60^ο^C; cooling: 30 s 40^ο^C.

To understand the gut microbiome dynamics in PD pathology, homozygous TG and control WT littermate rats were used in this study, which were obtained from heterozygous mothers to negate any maternal (genetic) effects on the microbiome analysis. First, heterozygous male (6–8 months (M) of age) was bred with 8–9 weeks old wild-type (WT) outbred SD female rats which were purchased from Charles River (F1). The pups born from the first outbred breeding pairs were genotyped and heterozygous (8 females and 4 males) from different outbred breeding pairs were set up for a second breeding (F2). All the pups were genotyped with standard PCR to detect the human SNCA gene to discriminate between WT and heterozygous rats. Heterozygous rats were again genotyped using q-RT-PCR to identify the DNA copy number for homozygosity at 3–4 weeks of age (one week for two rounds of genotyping – standard PCR for WT and heterozygous identification and second q-RT-PCR for homozygosity). Two cohorts were generated and used for the experiments. Cohort I—Microbiome dynamics of ageing: At the 4^th^ week, all the rats were separated into WT and homozygous TG groups (3–4 females/cage and males were kept based on genotyping results 2–4/cages if they were obtained from different breeding pairs) and were stocked separately from each other (major study). Cohort II – Co-housing group: both WT and homozygous rats were placed in the same cage since they were born (small study) or breeding maintenance. Furthermore, rats were also bred in two different facilities (Facility I and Facility II) with the same breeding plan for Cohort I study. Only heterozygous/homozygous males were transferred for breeding purposes and WT females were purchased from Charles River and used for generating cohorts for the microbiome study. The rats were housed 2–4 per cage under a 12 h light–dark cycle with ad libitum access to food and water. All experiments were performed according to the EU Animals Scientific Procedures Act and the German law for the welfare of animals. All procedures were approved (TVA: HG3/18) by the authorities of the state of Baden-Württemberg.

### Fecal sample collection for the microbiome dynamics study

Fecal pellets were collected from rats of different ages (4–5 weeks old rats to > 12 months) depending on the experiments for microbial diversity. To collect the fecal pellets, rats were removed from their home cages and individually placed into a new cage until they excreted fecal pellets (normally 2–3 pellets were collected). Within 5 min of excretion, fecal pellets were placed in cryotubes and transferred onto dry ice during the collection period. After all samples were collected, fecal pellets were stored at –80^o^C until use.

### Antibiotic cocktail treatment for rats

WT and TG rats (8–10 weeks of age) were orally administered a cocktail of broad-spectrum antibiotics – amoxicillin/clavulanic acid (4:1; 0.5 g/L), vancomycin (0.5 g/L), neomycin (0.5 g/L), cefuroxime (0.5 g/L), streptomycin (5 g/L), and ampicillin (0.5 g/L) in drinking water containing (5% sucrose). Rats were kept in individually ventilated cages (IVCs) one week before antibiotics were added to the drinking water (treatment starts) until the end of the experiments. Further, during the treatment period with antibiotics, rats were monitored twice per day for their health and safety (body weight, water content, and visual inspection). Each individual rat's body weight was measured. However, water intake (per cage; 100–150 mL of water with antibiotics/day depending on the number of rats in a cage) was measured for each cage rather than per individual rat. All antibiotic-treated rats remained healthy. A slight reduction of body weight (5–15% from the start days but later they gained some weight due to growth from the start) was observed. However, after one week, there was no difference in body weight between treated and untreated rats. Antibiotics were administered in an increasing dose (first 0.25 g/L after 3 days it was switched to 0.5 g/L for 2 weeks) in drinking water every alternate day. The reason for this was to acclimatize the rats to the antibiotics as of the initial concentration of 0.5 g/L, the rats did not drink the water and lost weight due to dehydration. Once antibiotics were given at the lower concentration (0.25 g/L) the rats began to drink and after 3 days, they were switched to a higher concentration 0.5 g/L antibiotic cocktail until the completion of the experiments.

### Bacterial DNA isolation

Bacterial DNA was isolated using the QIAamp FAST DNA stool Mini Kit (#51604, Qiagen, Germany) as described by manufacture’s recommendation. In brief, approximately 140–180 mg of fecal pellets were crushed and transferred into 2 mL tubes for bacterial DNA isolation (pathogen detection protocol was used). Samples were kept on ice until all feces were measured. After measurement, 1 mL of InhibitEX buffer was added to each tube containing fecal sample. The tubes were vortexed continuously until thoroughly homogenized and subsequently samples were kept at 70^o^C for 5 min and vortexed continuously for 15 s after the incubation. Samples were further centrifuged for full speed (12,000xg) for 1 min to pellet fecal particles. 200 µL of buffer AL was added and the samples vortexed for 15 s, incubated at 70^o^C for 10 min. After incubation, 200 µL of 100% ethanol was added to the lysate and mixed by vortexing. All the lysate was loaded onto QIAamp spin column and centrifuged at full speed for 1 min. Each column was then washed with 500 µL buffer AW1 and AW2. DNA was eluted in 100 µL of ATE buffer and stored at -20^o^C for further analysis.

### 16S rRNA sequencing and data analysis

To compare the diversity and composition of the gut microbial community, we isolated bacterial DNA from the feces of the WT and TG rats of the respective age (1 M—> 12 M) groups cohorts and performed PCR of the 16S ribosomal(r) RNA gene (variable region V3 and V4). The PCR products were sequenced using next generation sequencing. Sequenced amplicons were trimmed and analysed using bioinformatics tools.

For 16S rRNA gene amplification, 12.5 ng of DNA was amplified using 0.2 µM of both forward (TCGTCGGCAGCGTCAGATGTGTATAAGAGACAGCCTACGGGNGGVWGCAG) and reverse (GTCTCGTGGGCTCGGAGATGTGTATAAGAGACAGGACTACHVGGGTATCTAATCC; both from Metabion) and KAPA HiFi HotStart Ready Mix (#KK2601, KAPABiosystems). PCR was performed using a first denaturation of 95^o^C for 3 min, followed by 25 cycles of amplification at 95^o^C for 30 s, 55^o^C for 30 s and 72^o^C for 30 s, final elongation at 72^o^C for 5 min and the amplified DNA was stored at 4^o^C. DNA electrophoresis of samples was used to validate the amplicon specificity.

Samples were then subjected to Agencourt AMPure XP PCR purification system (Beckman Coulter) utilizing Agencourt’s solid-phase paramagnetic bead technology for high-throughput purification of PCR amplicons. Agencourt AMPure XP utilizes an optimized buffer to selectively bind PCR amplicons 100 bp and larger to paramagnetic beads. Excess primers, nucleotides, salts, and enzymes were removed using a simple washing procedure, resulting purified PCR product essentially free of contaminants. Further, PCR amplicons were indexed using Nextera XT DNA Library Prep Kit and KAPA HiFi HotStart ReadyMix. PCR was performed using first a denaturation of 95^o^C for 3 min, followed by 8 cycles of amplification at 95^o^C for 30 s, 55^o^C for 30 s, and 72^o^C for 30 s, and a final elongation at 72^o^C for 5 min. Samples were purified and then validated using BioAnalyzer (Bioanalyzer DNA1000, Agilent) and 4 nM of each library pooled using unique induces before sequencing on a MiSeq (Illumina) and paired 300 bp reads.

The obtained sequence reads were sorted by the unique sample barcodes, and these were subsequently removed along with the linker and primer sequences from the original sequencing reads using Fastx toolkit. Sequence reads were aligned using MALT (MEGAN alignment tool) for SILVA in semi-global mode and with percent identity threshold of 90%. Further analysis and visualization were performed using MEGAN-CE as described earlier [44].

### Shotgun sequencing

Isolated DNA was quantified using Qubit (Thermofisher) and presented comparable optical density quality ratio, concentration 26 – 75 ng/µL and fragment size distribution. Samples were diluted to 0.5 ng/µL with Tris (10 mM), and an input of 1 ng was processed following Illumina´s Nextera library preparation protocol. DNA was tagmented for 5 min at 55°C. The resulting DNA was then amplified and indexed using 12 PCR enrichment cycles and a dual combination of barcode primers. After amplification, DNA library clean-up was performed using AMPure XP bead purification and resuspended in resuspension buffer. Resulting libraries presented the similar molarity of 11 – 25 nM were pooled based on their molarity in hybridization buffer and denatured following the Nextera XT protocol. Libraries were sequenced using the Illumina NextSeq HighOutput using 2 × 150 bp paired end sequencing, providing 34 – 41 million clusters per sample. Data were analysed with MEGAN for abundance and metabolic gene pathways estimation was performed using eggNOG and InterPro2GO.

### Measurement of Na.^+^ Current and gut permeability using an ‘Ussing Chamber’

ENaC activity was estimated from the amiloride-sensitive potential difference and current across the rat colonic epithelium as described in mouse epithelium layer [45]. After removing the outer serosal and the muscular layer under a microscope, tissues were mounted onto a custom-made ‘mini-Ussing chamber’ with an opening diameter of 0.99 mm and an opening area of 0.00769 cm^2^. The serosal and luminal perfusate contained (in mM): 145 NaCl, 1 MgCl_2_, 2.6 Ca-gluconate, 0.4 KH_2_PO_4_, 1.6 K_2_HPO_4_, 5 glucose. To assess ENaC induced current, 50 µM amiloride (Sigma, in DMSO) was added to the luminal perfusate. ENaC activity was estimated from the effect of ENaC blocker amiloride (50 µM) on the transepithelial potential difference and current, which reflects the Na^+^ reabsorption from the colon epithelial layer. Transepithelial potential difference (Vte) was determined continuously and transepithelial resistance (Rte) estimated from the voltage deflections (ΔVte) elicited by imposing rectangular test currents of 1 µA and 1.2 s duration at a rate of 8/min. Rte was calculated according to Ohm's law.

### Preparation of the colon and brain for histological analysis

The colon/brain samples prepared on ice were fixed for 24 h in 4% PFA and then stored at 4^o^C in 0.4% PFA. Colon samples were prepared from rats deeply anesthetized by CO_2_ inhalation. Fixed colon samples were then alcohol-dehydrated and embedded in paraffin blocks using a tissue embedding station and stored at room temperature (RT). Paraffin blocks containing brains were cooled on ice block and cut in 7 μm thick sections using a microtome. Sections were placed in 43^o^C water bath for flattening, collected on a glass slide, dried in an incubator at 50^o^C for 1 hour (h) and stored at RT.

### Immunohistochemistry of the colon section

Sections were deparaffinized in xylene and rehydrated in decreasing ethanol. In some cases to identify insoluble α-Syn proteins aggregates, digestion of the colon tissues was performed with either Trypsin–EDTA (0.25%; #25200056, Thermofisher) containing phenol red (1:5 dilution) or 7–10 mg/ml of Proteinase K (#315836, Roche) in PK-buffer (10 mM Tris, 100 mM NaCl and 0.1% NP-40) at 37°C for 120 min. Heat antigen retrieval was performed for 15 min using a microwave set at 1000 W and using 10 mM sodium citrate (pH 6.0). Sodium citrate was refilled every 5 min to avoid the slides to drying out. Sections were then washed 3 times for 3 min in Tris buffered saline (TBS; Tris 1 M, NaCl 3 M and pH 7.5) and incubated 20 min in 0.3% hydrogen peroxide to block endogenous peroxidases. After washing the slides 3 times for 3 min in TBS, non-specific binding was blocked for 40 min by treating sections with 5% normal serum of the secondary antibody host animal (goat serum: S-1000, Vector; rabbit serum: S-5000, Vector; donkey serum: #017–000-001, Dianova) diluted in TBS and supplemented with 0.3% Triton X-100 to permeabilize the cell membranes. After washing the slides 3 times for 3 min with TBS, sections were incubated overnight with the primary antibody diluted in TBS containing 1% normal serum. In this study, we used anti-α-Syn (phospho S129) antibody (EP1536Y; 1:500 dilution) raised in rabbit (#ab51253, Abcam), anti-α-Syn antibody (LB508; 1:5000 dilution) raised in mouse (#ab27766, Abcam) and anti-α-Syn (#D37A7; 1:500) antibody specific to mouse/rat raised in rabbit (#4169, Cell Signalling). Next day, slides were washed 3 times for 3 min with TBS + 0.025% Triton X-100 and incubated for 1 h with a biotin conjugated secondary antibody raised in goat (mouse: #BA-9200, rabbit: #BA-1000, rat: BA-4000; Vector Laboratories) diluted 1:250 in phosphate buffered saline (PBS) containing 1% normal serum. Sections were washed 3 times for 3 min with TBS + 0.025% Triton X-100 and then incubated with an avidin–biotin enhancer complex coupled with peroxidase (ABC Elite; Vector) at RT for 1 h. After washing 3 times for 3 min with TBS + 0.025% Triton X-100, sections were detected using 3,3′-diaminobenzidine (DAB; # D12384, Sigma) providing a brown precipitate when oxidized by the peroxidase linked to the secondary antibody. The reaction was stopped by washing the slides in distilled water after 3 min. For microscopy, slides were mounted using a cover slip media, dried overnight, and stored at RT.

### Western blotting

The colons for the mass spectrometry or Immunoblotting analysis were collected from freshly euthanized rats and cleaned with PBS to remove faecal material and snap-frozen in liquid nitrogen and stored at –80^o^C until use. In certain experiments, myenteric plexus and epithelial layer were also isolated and samples were stored at –80^o^C until use.

Proteins were lysed from the colon tissues in 10 volume of RIPA buffer (50 mM Tris, 150 mM NaCl, 1.0% NP-40, 0.5% sodium deoxycholate, 0.1% SDS, pH 8.0) and supplemented with protease inhibitor (Complete; Roche Diagnostics) in order to perform gel electrophoresis. Brain or colon tissues were disrupted for 30 s using a homogenizer (T10 ultra turrax; VWR) in ice. After the homogenization, samples were incubated for 30 min at 4^o^C and spun for 20 min at 12,000xg. Protein lysate supernatants were supplemented with 10% glycerol before storage at -80^o^C.

Protein concentration was determined using the BCA method (BCA Protein Assay Kit; #23225, Life Technology) or Bradford assay. Samples were prepared by diluting protein lysates in PAGE buffer (0.2 M glycine, 25 mM Tris, 1% SDS), followed by a denaturation at 95^o^C for 10 min in loading buffer (80 mM Tris, 2% SDS, 5% 2-mercaptoethanol, 10% glycerol, 0.005% bromophenol blue, pH 6.8) and a short centrifugation for 30 s at 400xg. Proteins were separated by electrophoresis using 12% SDS-PAGE gel. Gels containing proteins were washed for 5 min in transfer buffer (0.2 M glycine, 25 mM Tris, 10–20% methanol) and transferred to membranes equilibrated in transfer buffer. Transfer was performed for 1 h 30 min at 80 V at 4^o^C on PVDF membranes. Immunoblots were washed for 5 min in TBS buffer and blocked using 5% non-fat milk (Slim Fast) in TBS. Membranes were then washed twice 5 min in Tris buffer saline containing 0.01% Triton X-100 (TBST) and incubated with the primary antibody over night at 4^0^C (human and mouse α-Syn: #610786 BD Biosciences). Membranes were then washed four times (5 min each) with TBST. Membranes were then incubated for 75 min with the secondary antibody coupled to horseradish peroxidase (GE Healthcare). After four washing steps with TBST (5 min each), bands were visualized using the enhanced chemiluminescence method (ECL + ; GE Healthcare). Light signals were detected using LI-COR Odyssey and quantified using Odyssey software.

### ELISA

To measure the calprotectin/MRP 8/14 from the fecal/serum samples, S100A8/S100A9 ELISA kit (#K6936, Immundiagnostik AG) was used according to the manufacture’s guidelines. First fecal samples were measured (weight 50 mg) and then dissolved in 500 μL of extraction buffer supplied by the kit, mixed by vortexing and then centrifuged for 10 min at 3,000xg. Supernatant was taken and transferred to a new microcentrifuge tube and 100 μL of sample was used for measuring the protein. The data were analyzed using 4 parameters algorithm and the concentration of calprotectin was normalized with feces weight and data presented in relative arbitrary units (A.U.).

### RNA-sequencing from the colon tissues

Briefly, 10 to 20 mg of frozen tissue was dissociated using 400 µL of RLT Plus in a 2 mL extraction tube containing a 5 mm diameter beads (Qiagen) and agitated at 30 HZ twice for 2 min in the TissueLyser II (Qiagen). Total RNA was extracted using QIAsymphony RNA kit (#931636, Qiagen). RNA isolation was performed on the QIAsymphony (Qiagen) following the platform Standard protocol. Elution was performed using 50 µL of RNase-free water.

RNA quality was assessed with an Agilent 2100 Bioanalyzer and the Agilent RNA 6000 Nano kit (Agilent). 3´RNA-sequencing was performed using 100 ng of total RNA and the QuantSeq 3' mRNA-Seq (#015.24; Lexogen, Austria). Libraries were sequenced on the NextSeq500 using the Mid Output v2.5 150 Cycles (#20024904, Illumina) with a depth of > 2 millions reads each.

FASTQ were generated using fastp (v0.20.0) and RNA-seq data quality assessed to identify potential issues with sequencing cycles, low average quality, adaptor contamination, or repetitive sequences from PCR amplification. Reads were aligned using STAR (v2.7.2a) against a custom-built genome composed of the Ensembl rattus norvegicus mRatBN7.2. Alignment quality was analyzed using MappingQC (v1.8) and visually inspected in the Integrative Genome Viewer (v2.4.19). Normalized read counts for all genes were obtained using edgeR (v3.26.6). Transcripts covered with less than 4 count-per-million in at least 1 sample were excluded from the analysis leaving > 14,000 genes for determining differential expression in each of the pair-wise comparisons between experimental groups.

RNA sequencing data was further subjected to Ingenuity pathway analysis (IPA) to identify the genes involved in different canonical pathways as well as with the reactome online tool.

### Flow cytometry for surface and intracellular staining

Plasma cells were first washed with PBS at 600xg for 5 min at 4^o^C. The supernatant was discarded, and cells were first stained with surface staining antibodies (CD4, CD8, CD25, CD44) then were fixed with Foxp3 fixation/permeabilization buffer (eBioscience, Germany) for intracellular staining and incubated for 30 min at RT. All the antibodies were purchased from eBioscience (nowThermofisher). After incubation, cells were washed with 1 × permeabilization buffer, exposed to added intracellular monoclonal antibodies (Foxp3, IL-17 and IFN-γ in different channel) and incubated for an additional 45 min at RT. Cells were washed again with permeabilization buffer and PBS was added. The cells were acquired on a flow cytometer (BD Fortessa™ from Becton Dickinson; Heidelberg, Germany).

### Whole-mount procedure and staining

Tunica muscularis strips were fixed with 4% (w/v) phosphate buffered paraformaldehyde (PFA; Sigma-Aldrich, St. Louis, MO, USA) for 20 min and rinsed three times with PBS. Antigen retrieval was carried out in citrate buffer (0.01 mol/l, pH 6.0, Roth, Karlsruhe, Germany) by heating the samples in a microwave (600 W for 5 min) and successively allowed the samples to cool. To prevent unspecific binding of antibodies, samples were blocked for two days at RT with PBS containing 4% (v/v) goat serum (Biochrom, Berlin, Germany), 0.1% (v/v) bovine serum albumin (BSA; Roth), 0.3% (v/v) Triton® X-100 (Roth), and 0.1% (wt/v) NaN_3_ (Merck, Darmstadt, Germany) on a shaker. Samples were then incubated with primary antibody diluted in PBS with 0.1% (v/v) BSA, 0.3% (v/v) Triton® X-100, and 0.1% (wt/v) NaN_3_ for three days at RT. Samples were then rinsed in PBS with 0.1% (wt/v) NaN_3_ three times, with the last washing step over night. Then secondary antibodies were diluted in PBS with 0.1% (v/v) BSA, 0.3% (v/v) Triton® X-100, and 0.1% (wt/v) NaN_3_ and were incubated overnight. Samples were then washed three times in PBS, immersed in 80% (v/v) glycerol (Sigma-Aldrich) in PBS for refractive index matching, and placed in a glass bottom petri dish for imaging.

The following primary antibodies were used for immunocytochemistry: mouse anti-α-Synuclein (1:500, BD Bioscience), rabbit anti-β-III tubulin (TUJ, 1:400, BioLegend, USA). Primary antibodies were detected using the fluorescent secondary antibodies goat anti-rabbit Alexa 488 (1:400, Invitrogen) and goat anti-mouse Alexa 546 (1:400, Invitrogen). Nuclear staining was carried out with DAPI solution (200 ng/ml, Roth). A Zeiss Axio Imager.Z1 fluorescence microscope with integrated Apotome module (Zeiss, Jena, Germany) was used for microscopic evaluation and structured illumination imaging. Images were acquired using Zeiss Zen blue software (Zeiss).

### Feces and serum sample preparation for the metabolite detection using ^1^H-NMR

For metabolite extraction and separation from lipids that disturb the proton nuclear magnetic resonance spectroscopy (^1^H-NMR) spectra of polar metabolites, a standard 2-phase extraction protocol was used. Here, 50 mg of deep-frozen feces sample were transferred into 2 mL adaptive focused acoustics (AFA) glass tubes (Covaris Inc, Woburn, USA) and mixed with 400 µL of ultrapure methanol and 800 µL of Methyl ter-butyl ether (MTBE; solvent grade). The mixture was manually dispersed with a disposable plastic spatula, then vortexed and transferred to a focused ultrasonicator (Covaris E220evolution, Woburn, USA). Feces metabolites were extracted with a 5 min lasting ultrasonication program in a degassed water bath at 7°C. After extraction, the metabolite suspension was separated into a polar and lipid phase by adding 400 µL of ultrapure water. In order to remove any remaining solids from the samples, the glass tubes were centrifuged for 5 min at 4,000xg. 700 µL of polar upper phase was then transferred to a fresh 1.5 mL eppendorf tube. The polar phase was subject to a 2nd centrifugation step for 5 min at 12,000xg and 600 µL of the supernatant transferred to a new 1.5 mL eppendorf tube and evaporated to dryness over night with a vacuum concentrator (Eppendorf Speedvac).

We prepared the serum samples for NMR analysis using following method. Briefly, 45 µL of serum and 90 µL of 100% methanol was used and then mixed with the Covaris 130 microTUBE glass vials for metabolites extractions (no phase separation was used). This procedure was done for 25 min (5 min/samples) in one cycle. Once the mixing was done, 135 µL of sample mixture supernatant was collected in a fresh 500 µL tube and subjected to centrifuge for 30 min at 12,000xg. After centrifugation, 120 µL of supernatant was taken in a fresh 1.5 mL Eppendorf tube and subjected to speed vacuum for overnight and the dried pellets dissolved in 50 µL NMR buffer (recipe + standards) was added and briefly sonicated to mix the buffer and metabolite pellets. Mixed solution 45 µL of the supernatant transferred with gel loading pipette tips into 1.7 mm NMR tubes (Bruker BioSpin, Karlsruhe, Germany) and a 96 well rack placed into the cooled (4°C) NMR autosampler.

For the feces samples, we used a slightly different protocol for NMR analysis. The dried fecal pellets (after overnight speedvac) were resuspended with 60 µl of deuterated phosphate buffer (200 mM K_2_HPO_4_, 200 µM NaN_3_, pH 7.4) containing 1 mM of the internal standard trimethylsilylpropanoic acid (TSP). For maximum dissolution, the plastic tubes were quickly sonicated and then centrifuged for 5 min at 14,000xg. 50 µL of the supernatant were transferred with gel loading pipette tips into 1.7 mm NMR tubes (Bruker BioSpin, Karlsruhe, Germany) and a 96 well rack placed into the cooled (4°C) NMR autosampler.

Spectra were recorded on a 600 MHz ultra-shielded NMR spectrometer (Avance III, Bruker BioSpin GmbH) equipped with a triple resonance (^1^H, ^13^C, ^31^P) 1.7 mm RT probe at 298 K. For optimum water suppression and shim adjustment a quick simple ZG experiment was performed followed by a 1 h lasting CPMG (Carr-Purcell-Meiboom-Gill) experiment in order to suppress residual background signals from macromolecules such as bilirubin (time domain = 64 k points, sweep width = 20 ppm, 512 scans). The recorded free induction decays (FIDs) were fourier-transformed and spectra phase- and baseline corrected (Bruker Topspin 3.5.6). The created files were processed with ChenomX NMR Suite 8.3 for metabolite annotation and quantification. For statistical analysis, different grouped metabolite concentration tables were created and imported with MetaboAnalyst 4.0. In detail, we investigated both the effect of ageing (between 3 and 14 M) as well as genotype (WT and TG) on the metabolic phenotypes in serum and feces.

### Cell culture, cytokine, and proteomic analysis for SH-SY5Y cells

SH-SY5Y neuroblastoma cells (passage number 14 to maximum 30) were cultured and maintained in T-75 cell culture flasks (CellSTAR^®^) with 20 ml of Dulbecco’s modified eagle’s media (DMEM) (Gibco^®^) with 10% FBS (Gibco^®^) and 1% antibiotic–antimycotic (Gibco^®^). For the 48 h treatment with succinate, cells were also cultured in a 1:1 ratio of F-12 Nut Mix (Gibco^®^) and Minimum Essential Medium (MEM) (Gibco^®^). The F-12 Nut mix media was supplemented with 10% FBS, 1% antibiotic–antimycotic and with MEM with 10% FBS, 1% antibiotic–antimycotic, 1 mM sodium pyruvate (Gibco^®^), 1,5 g/L sodium bicarbonate (Gibco^®^), and 1 × MEM non-essential amino acids (NEAA) (Gibco^®^). Flasks were kept at 37°C, 5% CO_2_, 95% humidity. Depending on the confluence, cells were either passaged or medium changed after 3 days. With 80% confluence, the medium was aspirated, and the cells were washed with 15 mL of Dulbecco’s PBS (Gibco^®^). After aspirating the PBS, 2 mL of Trypsin + 0,25% EDTA (Gibco^®^) was added onto the cells. The flask was then incubated for 5 min at 37°C, 5% CO_2_. The 2 mL of Trypsin was inactivated by adding 8 mL of DMEM or MEM/F12 and transferring the total of 10 mL from the flask to a 15 mL Falcon tube. After centrifuging the cell solution at 400xg for 5 min and discarding the supernatant, the pellet was resuspended in 10 mL of medium and split according to cell number. Cell counting was performed using an automated cell counter TC20^™^ (Bio-Rad) loaded with a 1:1 ratio of Trypan blue staining (0.4%) (Invitrogen^™^, Thermo Scientific) plus cell solution on a counting slide. New passage number flasks were set up with 2 × 10^6^ cells from the resuspended cell solution.

### Quantification of secreted cytokines

SH-SY5Y cells were seeded at a density of 400,000 cells/well in a 6-well plate and treated with 100 µg LPS, 50 µM, 100 µM, 200 µM, and 500 µM succinate and maintained under standard culturing condition. Untreated cells were applied as controls. After 48 h of stimulation, culture supernatant was harvested and IL-2, IL-6, IL-7, IL-8 (CXCL8), IL-10, CXCL10 (IP-10), CCL3 (MIP-1α), IL-1RA, CCL2 (MCP-1), G-CSF, IFN-α2, IFN-γ, TNF-α were measured using LEGENDPlex Multi-Analyte Flow Assay Kit (Biolegend). In this bead-based immunoassay assay, the beads are mixed into the harvested medium, and specific antibodies on the beads bind to the target proteins. The addition of detection antibodies and streptavidin–phycoerythrin produces unique fluorescent signal for each target protein with signal intensities proportional to the protein concentration. Fluorescent signals were measured using a flow cytometer and data analysis as per manufacturer’s recommendation.

### Immunofluorescence staining

SH-SH5Y cells were seeded in ibidi µ-Slide 8 Well high glass chambers and treated with 50 µM, 100 µM, 200 µM, and 500 µM, succinate and maintained under standard culturing condition for 24–48 h. Media was removed and cells were fixed with 100% chilled methanol for 10 min on ice. After, fixation, cells were washed 3 × for 5 min in PBS to remove fixation solution. To minimize intra or extracellular background signals, non-specific antigens were blocked by incubating the fixed cells with 10% normal goat serum (NGS) in PBS for 1 h. Cells were washed 3 × for 5 min in PBS. Then cells were incubated with 1:200 diluted succinate receptor 1 (SUCNR1 or GPR91 #PA5-99450; Thermofisher) antibody in PBS containing 1% NGS for overnight at 4^o^C. After primary antibody incubation, cells were washed again 3 × for 5 min in PBS and performed secondary antibody (Goat anti-Rabbit IgG (H + L) Cross-Adsorbed Secondary Antibody, Alexa Fluor™ 488; #A-11008, Thermofisher) staining 1:500 dilution in PBS containing 1% NGS for 1 h. Cells were washed again 3 × for 5 min in PBS. DAPI containing antifade medium was used for nuclear staining and EVOS fluorescence microscope was used for acquiring the images.

### In-gel protein digestion

SH-SY5Y cells were seeded at a density of 400,000 cells/well in a 6-well plate and treated with 100 µg LPS, 100 µM succinate, and 100 µg LPS + 100 µM succinate for 48 h in four biological replicates. Protein pellets were purified with SDS-PAGE (Thermo Fisher Scientific). Coomassie-stained gel pieces were excised, and in-gel digested using trypsin. Extracted peptides were desalted using C18-StageTips and proceeded to liquid chromatography and tandem mass spectrometry (LC–MS/MS) analysis.

### Mass spectrometry (LC-MS/MS) for protein detection

LC–MS/MS analysis was conducted on an Easy nano-LC (Thermo Fisher Scientific) coupled to an QExactiveHF mass spectrometer (Thermo Fisher Scientific). Peptides were eluted applying a 60-min segmented gradient at a flow rate of 200 nl/min, selecting 20 most intensive peaks for fragmentation with HCD.

### Mass spectrometry (LC-MS/MS) data processing

The MS data from all replicates were processed together using MaxQuant software suite v.1.6.7.0. Database search was performed using the Andromeda search engine, which is integrated in MaxQuant. MS/MS spectra were searched against a target-decoy human Uniprot database consisting of 96,817 protein entries and 245 commonly observed contaminants. In the database search, full specificity was required for trypsin. Up to two missed cleavages were allowed. Carbamidomethylation of cysteine was set as fixed modification, whereas oxidation of methionine and acetylation of protein N-terminuns were set as variable modifications. Initial mass tolerance was set to 4.5 parts per million (ppm) for precursor ions and 0.5 dalton (Da) for fragment ions. Peptide, protein and modification site identifications were reported at a false discovery rate (FDR) of 0.01, estimated by the target/decoy approach. Label-free algorithm was enabled, as was the “match between runs” option for samples within one biological replicate. Label-free quantification (LFQ) protein intensities from the MaxQuant data output were used for relative protein quantification. Downstream bioinformatic analysis (two-samples Student's t-tests and Volcano plots) was performed using the Perseus software package, version 1.6.2.3. Data was filtered for contaminants, reverse and only identified by site entries. Two samples Student t-tests were performed, considering *p* < 0.05 to be statistically significant and setting SO = 0. Volcano plots were generated with FDR of 0.01, number for randomization was set to 250.

### Statistical analysis

MEGAN-CE (version 6.14.2, built 23 Jan 2019) and MicrobiomeAnalyst were used for data acquisition and analysis. GraphPad and Inkscape were used for the final figure preparation. One-way analysis of variance (ANOVA), Two-way ANOVA (post-hoc Sidak’s test) or Student’s t-test was used for statistical analysis using GraphPad (version 8/9) wherever it was appropriate and described in the figure legend. Data shown in violin plots which represents the median and quartiles as the box-and-whisker plots and it also displays a smoothed frequency distribution. The *p* value (≤ 0.05) was considered significant. Metabolite concentrations from ^1^H-NMR analysis were exported as comma separated value spreadsheet file to MetaboAnalyst, normalized with PQN (probabilistic quantile normalization) to account for dilution effects and pareto scaled for making metabolites within one sample comparable. A combined fold change (FC > 1.2) and Student’s t-test analysis (*p* ≤ 0.05) was used for the volcano and box plots. Furthermore, multivariate heat maps, partial least squares-discriminant analysis (PLS-DA) plots and variable importance in projection (VIP) scores were generated for direct comparison of the genotypes at 3 M and > 12 M in feces and serum, respectively. The effect of antibiotics treatment on serum and feces metabolite profiles was investigated by heat maps, sparse PLS discriminant analysis (sPLS-DA) and VIP scores. The correlation analysis between metabolites (serum and feces) and bacterial abundance was performed using “pysch” and “corrplot” packages in R Studio (4.1.2).

## Results

### Microbiome dynamics in the (α-Syn) TG rats

**Fig. 1:**
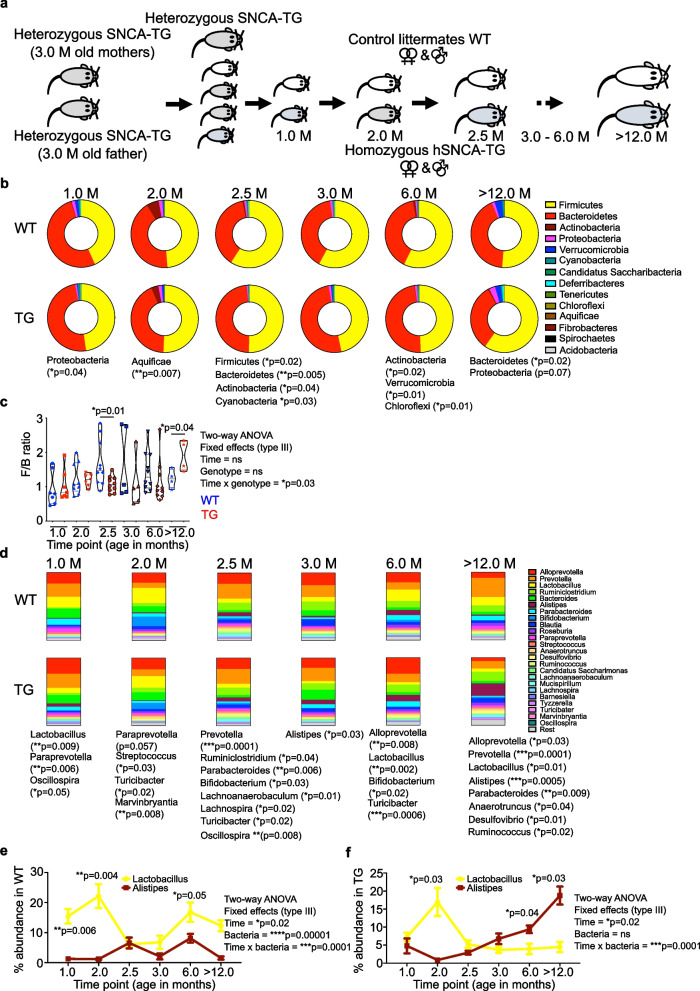
Gut microbiome dynamics with ageing, decreased *Lactobacillus* and increased *Alistipes* bacterial genera in the BAC-hSNCA (α-Syn) TG rats. **a** To understand the microbiome dynamics with ageing, heterozygous females and heterozygous males were used for breeding to keep the same gut microbiome from the mother to avoid maternal effect. When females were pregnant, the male was removed from the cage and pups were allowed to stay with the mothers for 3 weeks or until weaning. Three weeks old pups were genotyped and then separated into either WT or homozygous TG groups then fecal sample collections were started at the age of 1 M (4–5 weeks age 1 M; *N* = 7 WT (female) and *N* = 7 TG (female)) onwards [2 M; *N* = 8 WT (4 male and 4 female) and *N* = 7 TG (4 male and 4 female), 2.5 M; *N* = 10 WT (6 male and 4 female) and *N* = 10 TG (6 male and 4 female), 3 M; *N* = 5 WT (female) and *N* = 5 TG (female), 6 M; *N* = 10 WT (6 male and 4 female) and *N* = 10 TG (6 male and 4 female), > 12 M (12–14 M); *N* = 4 WT (male) and *N* = 4 TG (male)] for the microbiome analysis by either 16 s rRNA gene amplicon or shotgun sequencing methods. The animals were kept in 3–4 different cages to avoid the ‘cage affect’ bias in data analysis. Both male and females were used for the fecal sample collection depending on the breeding. **b** Representation of bacterial diversity (hollow pie chart) at phylum levels in WT and TG rats. **c** F/B ratio was calculated for WT and TG rat samples and significant difference was observed at 2.5 M and > 12 M age. F/B ratio was lower in TG rats at 2.5 M of age however, this ratio was reversed at > 12 M of age which was significantly higher in TG rats. Student’s t-test was performed for comparisons at a given age for WT and TG. Two-way ANOVA was performed to find the significance between different time points and genotypes. **d** The microbiome dynamics at genera level was estimated. Each bar chart represents the % relative abundance of bacterial genera of the total bacteria in each group at particular age. Significant change in bacterial phyla is shown in asterisk for particular phyla together with *p* value significance. **e**, **f** The dynamic representation of *Lactobacillus* and *Alistipes* with ageing in WT and TG respectively. A significant difference was observed with ageing in *Lactobacillus* and *Alistipes*. Two-way ANOVA was performed to find the significance between different time points and genotypes. The plots show the means ± SEM at each time point (age in months) with respective numbers shown in (**a**). *P* value significance represents *p ≤ 0.05, **p ≤ 0.01 and ***p ≤ 0.001

To determine the effect of human α-Syn overexpression on the gut microbiome’s composition and diversity, we analysed fecal pellets from the colon of the WT and TG rats by 16S rRNA gene amplicon sequencing. We used homozygous TG and control WT littermate rats, which were obtained from heterozygous mothers (Fig. 1a; a detailed description of breeding and genotyping is provided in Materials and Methods section) to negate any maternal (genetic) effects on microbiome analysis. The total numbers of reads between both WT and TG rat samples for each respective age group was shown for our sequencing depth (Suppl. Figure 1a). Alpha diversity captures both the organismal richness of a sample and the evenness of the organisms’ abundance distribution [46, 47]. We estimated the population diversity of the microbial community (α-diversity) by Chao 1 and Shannon–Weaver index with the MicrobiomeAnalyst tool [48] and MEGAN-CE software [49] at phylum level. The - α-diversity Shannon–Weaver index in TG rats tended to be lower at each time point at phylum level, a trend, however, not reaching significance (Suppl. Figure 1b). Most of the bacteria belonged to the Firmicutes and Bacteroidetes phyla, and both are typically the dominant phyla in the gut microbiome in humans and animals (Fig. 1b). At each age group, only a few bacterial phyla were significantly altered (WT vs TG comparisons), but most significant changes were observed during 1 M (Proteobacteria; *p* = 0.04), 2 M (Aquificae; *p* = 0.007) 2.5 M (Firmicutes; *p* = 0.02, Bacteroidetes; *p* = 0.005, Actinobacteria; *p* = 0.04 and Cyanobacteria; *p* = 0.03), 6 M (Actinobacteria; *p* = 0.02, Verrucomicrobia; *p* = 0.01 and Chloroflexi; *p* = 0.01) and > 12 M (12-14 M) (Bacteroidetes; *p* = 0.02) of age in TG rats (Fig. 1b and Suppl. Figure 1c). Further, with ageing the Bacteroidetes phylum was significantly reduced in TG rats (Fig. 1b and Suppl. Figure 1c). The Proteobacteria phylum abundance was increased in > 12 M TG rats, however, it did not reach a significant level (Fig. 1b). Further, the Firmicutes and Bacteroidetes (F/B) ratio was calculated for the respective age group in WT and TG rats. The 2.5 M age group had a significant reduction in F/B ratio in the TG group (Fig. 1c). In sharp contrast, this ratio was significantly increased in  > 12 M old TG group (Fig. 1c). Further, we performed Two-way ANOVA to find a correlation between change in the F/B ratio affected by age and bacteria. Our analysis suggested that with age there is a change in F/B ratio but both factors independently are not correlated (Fig. 1c). Additionally, we analysed the - α-diversities (Shannon–Weaver index and Chao 1) (Suppl. Figure 1d) and the bacterial composition at the genus level (Fig. 1d). Surprisingly, even at an early age (2.5 M), we were able to detect bacterial dysbiosis in TG rats compared to WT control rats and observed that ageing accelerates bacterial dysbiosis dynamics significantly (Fig. 1d and Suppl. Figure 1e and Suppl. Figure 2). The most interesting correlation was between *Lactobacillus* and *Alistipes*. With ageing TG rats had decreased relative abundance of *Lactobacillus* whereas the relative abundance of *Alistipes* genus was significantly increased (Fig. 1e, f). Two-way ANOVA suggested that in WT and TG rats, with age there is change in the dynamics of *Alistipes* and *Lactobacillus* (Fig. 1e & f). Furthermore, gender-based data analysis at 6 M age suggested that both gender have reduced *Lactobacillus* and *Turicibacter* abundance (Suppl. Figure 1f & g). Thus, overall, bacterial composition at genera level was significantly different between WT and TG rat at any given age group (Suppl. Figure 2).

Several patients and murine model studies have suggested that *Lactobacillus* is abundantly present in relation to PD [19, 25, 26, 29, 50]. In contrast, other studies including ours suggested that *Lactobacillus* is decreased in PD models as well as in PD patients [20, 31, 44]. To understand the discrepancies in the *Lactobacillus* abundance with respect to α-Syn overexpression, we performed a mother to children microbiome follow up study. Microbiome composition analyses suggested that at 2 M of age, WT and TG rats have a different pattern of *Lactobacillus sp*. in their feces (Suppl. Figure 3a, b, & c). Additionally, there was no detectable effect of the cages on the microbiome composition (Suppl. Figure 3a).

Different environments as found in different animal facilities could change the gut microbiome. Therefore, both WT and TG rats were housed in two separate animal facilities (Facility I and Facility II) and their gut microbiome composition was investigated. We found that animal housed in two separate animal facilities present a similar bacterial composition including *Lactobacillus* abundance (Suppl. Figure 4a & b). However, *Lactobacillus sp*. abundance varied in TG rats, although the trend was less *Lactobacillus* compared with WT (Suppl. Figure 4b).

Next, we investigated whether keeping both genotype rats in the same cage, could change the gut microbiome of older TG rats (‘cage transfer transplant’) or whether `genetic factors` were still predominant in determining the microbiome. Thus, we performed a pivotal experiment to answer this conjecture. Firstly, WT and TG (4 females only) rats were kept separately for > 12 M and at the end of the experimental period faecal pellets were collected (24 h prior) (Suppl. Figure 5a). Subsequently, the groups were then divided (2 WT and 2 TG) and kept together in fresh cage for one week and faecal pellets collected at the beginning (day 0) and end of the experiment (day 7). We found that *Lactobacillus* remained reduced whereas *Alistipes* continued to be elevated (Suppl. Figure 5e & f). The ratio of *Lactobacillus* and *Alistipes* was not changed significantly by the cage transfer (Suppl. Figure 5). Furthermore, WT and TG rats were also kept for 12 months in the same cages since birth, we found that *Lactobacillus* abundance was still lower, however, it did not reach a significant level (Suppl. Figure 5g). Thus, overall, we concluded that α-Syn overexpression could hold a key role in governing the expression of different bacterial genera including *Lactobacillus*.

### Metagenomic bacterial functional analysis in older TG rats

**Fig. 2:**
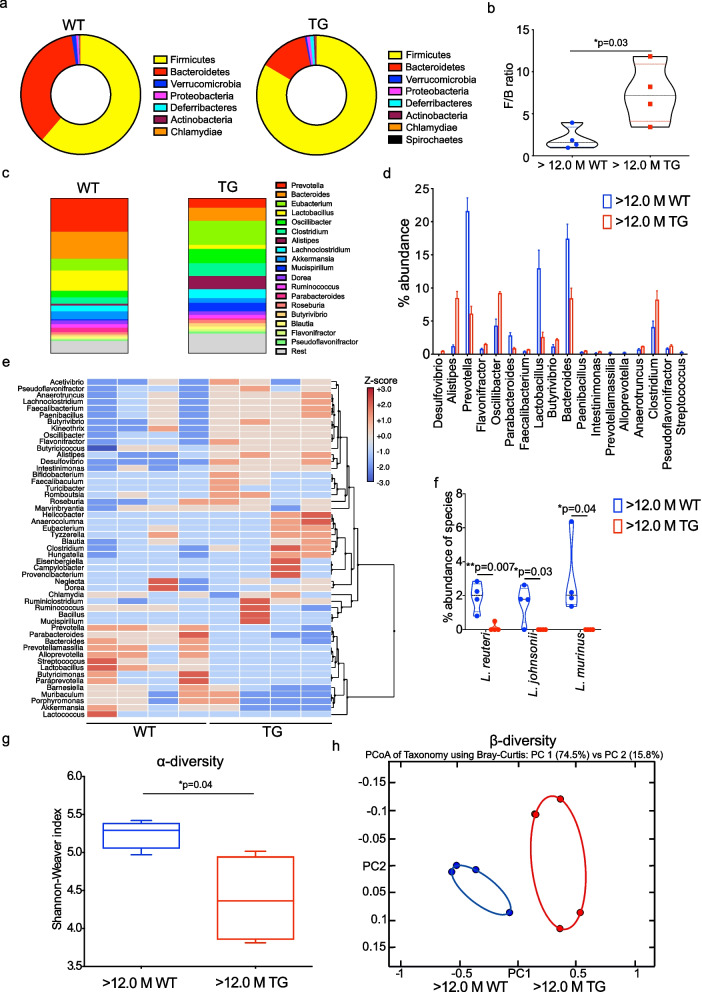
Gut dysbiosis in ageing TG rats. **a** To find out the bacterial dysbiotic bacterial strains, we have performed shotgun DNA sequencing and calculated the abundance of bacterial phyla (hollow pie charts) at > 12 M; *N* = 4 WT (male) and *N* = 4 TG (male). **b** F/B ratio was significantly higher in TG compared with WT. **c**, **d** Several bacterial genera were significantly upregulated (*Desulfovibrio, Alistipes, Flavonifractor, Oscillibacter, Paenibacillus, Clostridium* and *Butyrivibrio*) and significantly downregulated (*Streptococcus, Bacteroidetes, Lactobacillus, Parabacteroidetes*, and *Prevotella*) at > 12 M age in TG rats. **e** Clustering of gut bacteria into three major clusters from WT and TG rats. **f** Abundance of *Lactobacillus* species in WT and TG rats. **g** α-diversity at genera level calculated using Shannon–Weaver index and found to have significantly lower diversity in TG rats compared with WT. **h** β-diversity estimated for both the genotypes of rats with Principal coordinate analysis (PCoA) using Bray–Curtis method and both WT and TG rats cluster differently (PC1 54.5% and PC2 15.8). *P* value significance represents *p ≤ 0.05

The microbial dysbiosis with ageing in TG rats (> 12 M) was observed by 16S rRNA gene amplicons sequencing. However, this method is limited as it relies on the amplification of a single gene using PCR-based of confined regions of the bacterial genome only. To increase the sensitivity of the microbiome sequencing, we performed shotgun sequencing which also allows sequencing of the whole bacterial genome and also provides critical metabolic function interaction of the bacterial community. The shotgun sequencing depth and taxonomic rarefaction shown in Suppl. Figure 6 for both WT and TG rats. Again, the F/B ratio was significantly higher in TG compared with control WT littermates at phylum level (Fig. 2a, b). The α-diversity was significantly reduced in TG rats (Fig. 2). Furthermore, based on genus level taxonomic unit, we calculated the percentage (%) composition of the gut bacteria in WT and TG rats and found that *Lactobacillus* was reduced in > 12 M TG rats compared with WT, whereas *Alistipes* was significantly increased in the TG rats (Fig. 2c, d, e & Suppl. Figure 6). Composition of *Lactobacillus* strains *L. reuteri, L. johnsonii, L. murinus* was significantly lower in TG rats (Fig. 2f). α and -β- diversities represent the similarity (or difference) in organismal composition within and in between the samples. α-diversity was measured using the Shannon–Weaver index revealing that α-diversity was significantly higher in WT compared with TG rats (Fig. 2g). Next, using the principal component analysis (Bray–Curtis method) to investigate β-diversity, we found that the gut microbiome at the genus level from WT and TG rats cluster separately (Fig. 2h). Functional analysis of the bacterial genome revealed that several pathways related with metabolic activities were identified and dysregulated (Suppl. Figure 7). Overall, our data indicate that α-Syn overexpression induces the gut microbiome dysbiosis which could alter metabolite production in ageing TG rats.

### TG rats have a dysregulated metabolite production

**Fig. 3:**
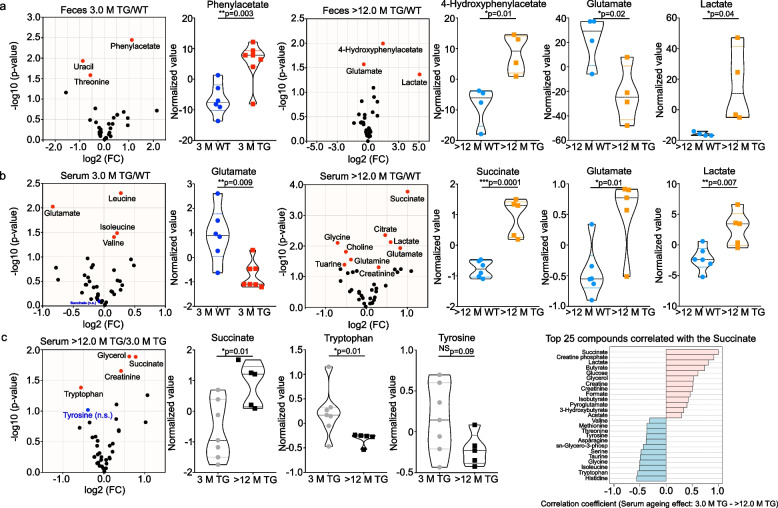
Ageing affects the succinate, tryptophan and tyrosine metabolites in the feces and serum. Metabolites combined fold change (FC > 1.2 (log2 FC)) and *p*-value < 0.05 (-log10 (*p* value)) volcano plot analysis identified effect of ageing between WT and TG rat feces and serum. **a** At 3 M age feces genotype comparison TG/WT phenylacetate is significantly upregulated whereas at > 12 M age 4-Hydroxyphenylacetate and lactate were up while glutamate were down. **b** In serum samples glutamate is significantly decreased at 3 M whereas at > 12 M succinate, glutamate and lactate are increased. **c** When only observing the ageing effect on TG serum metabolites, succinate is significantly upregulated, and succinate correlation coefficient analysis suggested that tryptophan and tyrosine were negatively correlated with succinate while other short chain fatty acids were positively correlated. Statistically significant levels showed in the respective volcano and violin plots. *P* value significance represents *p ≤ 0.05, **p ≤ 0.01 and ***p ≤ 0.001

Based on bacterial genome (eggNOG and InterPro2GO) metabolic analysis, we identified that N-formylglutamate deformylase (EC 3.5.1.68) pathway gene, which is involved in formate and L-glutamate production, was significantly downregulated in TG. Whilst, succinylglutamate desuccinylase (EC 3.5.1.96) pathway gene which is involved in succinate and L-glutamate production, was unregulated in TG (Suppl. Figure 6e & f). To further understand how changes in the bacterial composition could lead to different metabolites production at functional level, we performed ^1^H-NMR-based metabolomics from faecal and serum samples of younger (3 M) and older (> 12 M) WT and TG rats (Fig. 3a). In total, we were able to quantify 31 different metabolites from feces and 38 metabolites from serum (Suppl. Figure 7). Multivariate statistics PLS-DA was employed to identify differences between WT and TG rats for each age group (3 M and > 12 M) for the feces (Suppl. Figure 7a, b) and serum samples (Suppl. Figure 7c, d). At > 12 M of age, there was a clear separation between WT and TG rat samples (Suppl. Figure 7b, d). However, this gap was less discrete for 3 M age group samples (Suppl. Figure 7a, c). When comparing VIP scores of the PLS-DA feces analysis, we identified the most important metabolites—high levels of succinate in 3 M TG rats but high levels of glutamate were discerned in > 12 M WT rats (Suppl. Figure 7a, b). In serum, 3 M WT rats showed elevated glucose levels, whilst > 12 M TG rats showed high lactate and succinate levels (Suppl. Figure 7c, d).

To support the findings of multivariate statistics we applied a stringent univariate volcano plot analysis (combination of fold change and Student's t-test). We compared the metabolite levels based on genotype at each age group (3 M and > 12 M) where comparisons were made for TG *vs* WT (TG/WT) (Fig. 3a, b) in the feces and serum respectively. Further, we compared the levels of the metabolites for each genotype with age (3 M to > 12 M) for WT (Suppl. Figure 8a) and TG (Fig. 3c). We used FC > 1.2 (log2 FC) and *p*-value < 0.05 (-log10 (*p* value)) by default for all the metabolites and plotted against both values as volcano plots (Fig. 3 & Suppl. Figure 8). At 3 M of age, feces genotype comparison TG/WT suggested that phenylacetate was significantly upregulated whereas at > 12 M of age (TG/WT) a divergent set of metabolites such as 4-Hydroxyphenylacetate and lactate were upregulated while glutamate was significantly downregulated (Fig. 3a). We identified the most significant changes in the serum samples from > 12 M old TG and WT rats. We observed very high levels of lactate and succinate in TG compared to WT (Fig. 3b). Interestingly, when we explored serum metabolites, we found that glutamate was significantly downregulated at a younger age (3 M). In contrast with ageing, succinate, glutamate and lactate were significantly upregulated in older (> 12 M) TG rats compared to WT (TG/WT comparisons at a given age) (Fig. 3b). Further, we performed the ageing comparison for each genotype either WT or TG and found that serum succinate in TG rats was significantly upregulated (Fig. 3c; left). Succinate correlation coefficient analysis suggested that tryptophan and tyrosine were negatively correlated with succinate while other SCFAs were positively correlated (Fig. 3c; right). In WT serum succinate tended to decline with ageing, a trend, however, not reaching statistical significance (Suppl. Figure 8a). Whilst observing the ageing effect for feces, the correlation analysis with succinate suggested tyrosine is negatively correlated, whilst 4-Hydroxyphenylacetate was positively correlated in TG rats (Suppl. Figure 8b).

**Fig. 4:**
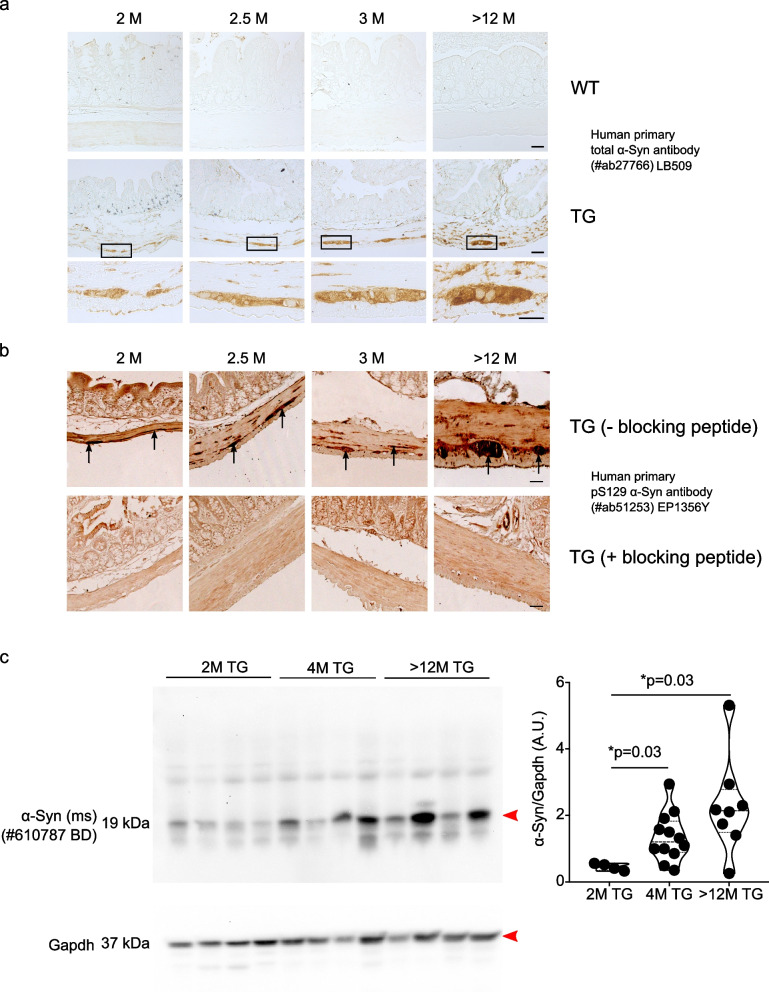
Accumulation of α-Syn in the colon of TG rats. **a** IHC was performed to identify the accumulation of total human α-Syn in 2, 2.5, 3 and >12 M old WT and TG rats using human primary α-Syn antibody (LB509; #ab27766). With ageing accumulation of α-Syn was observed in TG rats whereas no positive staining was observed in WT rats as expected. **b** Further, pathological state of human α-Syn was examined in 2, 2.5, 3 and > 12 M in TG rats using human primary pS129 α-Syn antibody (EP1356Y; #ab51253). It appeared that pathological α-Syn was increased with ageing in TG rats. Further, pS129 α-Syn antibody was blocked using specific peptide for pS129 site for the antibody and no staining was observed suggesting the specificity of pS129 antibody. **c** One of the representative immunoblot images of α-Syn and Gapdh staining is shown. We quantified the α-Syn amount using total α-Syn antibody (#BD 610787) and found that with ageing (2 M) to 4 M α-Syn expression in the colon tissues is significantly increased. Further ageing (4 M to > 12 M) tended to affect the accumulation of α-Syn but not statistically significant difference was observed. *P* value significance represents *p ≤ 0.05

To understand the functional correlation between bacterial abundance and metabolites, we performed Pearson correlation analysis and identified that succinate levels in the feces were positively and significantly correlated with *Blautia, Desulfovibrio* and *Tyzzerella* and glutamate with *Parabacteroides* abundance (Suppl. Figure 9a, c). The most interesting positive correlation was observed for 4-Hydroxyphenylacetate with *Alistipes* whilst *Lactobacillus* was negatively correlated (Suppl. Figure 9). Earlier, it was reported that *Alistipes indistinctus, paraprevotella* strains *clara & xylaniphila, Blautia wexlerae,* and *Akkermensia muciniphila* could be a succinate producing bacteria [51, 52]. Further, a recent report suggested that succinic acid levels in the plasma of PD patients is correlated with constipation [53]. Our correlation analysis for serum metabolite with bacterial phyla revealed that *Alistipes* was also positively correlated with succinate levels (Suppl. Figure 9b). Overall, it appears that dysregulated levels of succinate, lactate and tyrosine could be linked with PD progression and dysregulated microbiome levels.

### Presence of synucleinopathy in the enteric nervous system (ENS) of TG rats

Using antibodies specific for human α-Syn protein, we detected using immunohistochemistry human specific staining of the α-Syn in the colon of TG rats. We found presence of human α-Syn expression in the gut of TG rats whereas we did not detect any human α-Syn expression in WT rats (Fig. 4a). Human α-Syn staining was mostly found in the ENS of the colon and also in the myenteric plexus of TG rats. Presence of the transgenic human α-Syn protein was also validated by immunoblotting (Suppl. Figure 10a). Expression of the human α-Syn expression was also detected in the myenteric plexus and in the colon of TG rats using whole-mount immunostainings of the Tunica muscularis (Supp. Figure 10b).

To investigate the presence of potential pathological forms of α-Syn and an accumulation of α-Syn in TG rats, we first performed the histological analysis of the colon. We could detect reproducibly, the presence of phosphorylation of human α-Syn at serine 129 position (human-specific α-Syn antibody for transgenic expression) which increased with ageing (Fig. 4b). Presence of synucleinopathy was investigated using trypsin/Proteinase K digestion to identify the insoluble forms of α-Syn protein. TG rats exhibited positive staining for total endogenous rat/human α-Syn and pS129 α-Syn after trypsin digestion and total endogenous rat/human α-Syn after Proteinase K digestion even in young rats suggesting that aggregation of α-Syn already occurs at two months of age (Suppl. Figure 11). Further, total accumulation of human α-Syn increased with age in TG rats (2 M) and reached a plateau at the age of 4 M (Fig. 4c). At later stages (> 12 M), α-Syn still tended to increase slightly, but no significant change to 4 M age was observed (Fig. 4c). Together, our data suggest that human α-Syn accumulation occurs in the colon of young TG rats.

The prominent presence of C-terminally α-Syn truncated fragments was previously detected in the brain of TG rats. [40]. To test this, we performed immunoblotting of human α-Syn from the colon of TG rats and identified several small-sized fragments of α-Syn in different age groups (5 M and  > 12 M) (Suppl. Figure 12a). We found 3 major truncated fragments of human α-Syn protein which we characterized further using specific α-Syn antibodies targeted at N- or C-terminal epitopes (Suppl. Figure 12c). Truncated fragments did not arise from the boiling of the proteins as samples prepared in the presence of Disuccinimido dithiobispropionate; Di(N-succinimidyl) 3,3'-dithiodipropionate (DSP; a cross-linker) led to the same results (Supp. Figure 12b). Interestingly, α-Syn exhibited two N-terminal truncation fragments and one C-terminal truncation fragment (Suppl. Figure 12c). Thus, α-Syn expression in the colon revealed in addition to brain pathology that intestinal tissue is valuable in assessing PD progression in TG rats.

### Physiological functions of the TG rats are dysregulated with ageing

**Fig. 5:**
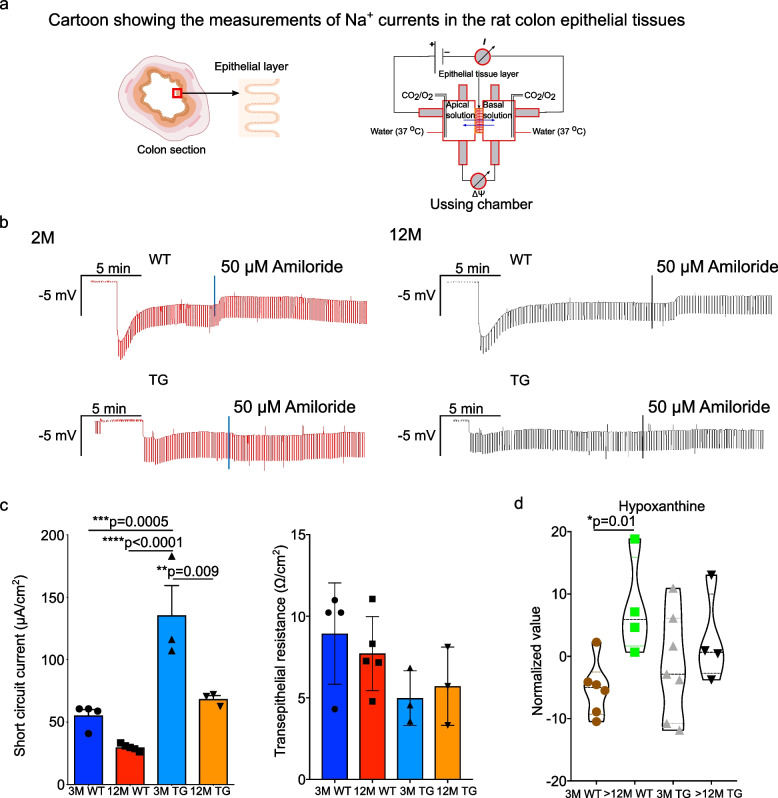
Increased transepithelial current in TG rats. **a** A schematic diagram showing the ‘Ussing chamber’ technique to measure the function of intestinal permeability and Na ^+^ uptake to gut epithelial cells (ENaC channel). **b** Original tracing illustrating the effect of currents (1 µA) and of amiloride (50 µM) on transepithelial potential across colonic epithelium from WT (upper) and TG (lower) from 2 and 12 M rats (*N* = 3–5 female/genotype). **c** Arithmetic mean ± SEM (*N* = 3–5/group) of amiloride-sensitive current across colonic epithelium (Na^+^ absorption) from TG and WT rats and resistance of colonic epithelium (permeability). **d** Fecal hypoxanthine levels measured by ^1^H-NMR from 3 M and > 12 M WT and TG rats. *P* value significance represents *p ≤ 0.05, **p ≤ 0.01, ***p ≤ 0.001 and ***p ≤ 0.001

The colon is a significant site of water and salt absorption, and during this process it desiccates the feces. The dysregulation in the gut microbiome and metabolites could influence the intestinal permeability. Therefore, we used an Ussing chamber to evaluate the function of intestinal permeability and Na^+^ uptake. To do so, we measured the transepithelial Na^+^ current (ENaC) in the colon epithelium. We measured the ENaC current in young (2 M) and older (12 M) rats of both the genotypes and found that 2 M and 12 M TG rats had a lower ENaC current compared with WT rats respectively (Fig. 5a, b, and c). This could suggest that TG rats may exhibit a dysregulation of the Na^+^ absorption in the colon. Further, we found that the transepithelial resistance tended to decrease with ageing. However, no significant change was observed between WT and TG at any age group (Fig. 5c). Interestingly, the feces’ water content showed no difference between 12 M WT and TG rats (data not shown). Hypoxanthine is negatively correlated with intestinal inflammation and permeability in inflammatory bowel disease [54]. We found that hypoxanthine is significantly increased in > 12 M WT compared with 3 M WT rats but not in TG rats (between 3 M and > 12 M). But, hypoxanthine was not significant between TG and WT rats (Fig. 5d). Nonetheless, gut dysbiotic metabolites could affect the Na^+^ uptake in TG rats.

### Increased local and systemic inflammation in TG rats

**Fig. 6:**
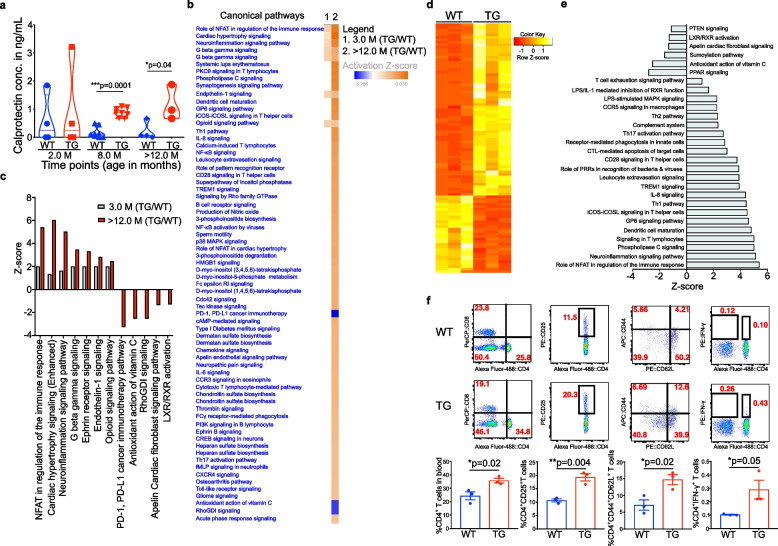
Increased inflammatory signals in the colon MS tissue layer of > 12 M TG rats. **a** Fecal/serum calprotectin levels measured by ELISA in 2, 8 (fecal) and > 12 M (serum; s) WT and TG rats. **b** WT and TG (3 M and > 12 M old) rat colon MS tissue layer or gut epithelium subjected to RNA-seq. **b**, **c** IPA from TG and WT rats showed upregulation of several biological pathways in > 12 M TG rats. **d** The heatmap shows upregulation (yellow) and downregulation (red) genes in the gut MS tissue layer. TG rats have higher expression of various genes involved in inflammation compared with WT. **e** IPA analysis in > 12 M TG rats suggested that several inflammatory pathways were changed. (f) FACS plots show increase in numbers of CD4^+^ T cells, CD4^+^CD25^+^ T cells (activated), CD^+^CD44^+^ T cells (memory) and CD4^+^ IFN-γ^+^ T cells were significantly higher in the blood of > 12 M TG rats compared with WT. Bar plots show the means ± SEM. *P* value significance represents *p ≤ 0.05

PD patients have a higher abundance of inflammatory proteins in the feces, possibly be due to dysbiotic changes in the gut microbiome [55–57]. Thus, we measured the fecal/serum inflammatory proteins in TG rats. Calprotectin levels were significantly increased in older fecal (8 M) and serum (>> 12 M) TG rats compared with WT, however, no change occurred at an earlier age (2 M; fecal samples) (Fig. 6a). To determine where the sources of these increased inflammatory proteins in the feces, we extended our evaluation on the gene expression in the host intestine. Thus, we performed RNA-sequencing (RNA-seq) from the mucosa and submucosal tissues (defined as MS tissue layer) from young (3 M) and older (> 12 M) TG and control WT rats. Our RNA-seq experiments suggested that an enrichment of dysregulated genes involved in inflammation was detected with ageing in TG rats (Fig. 6b-e and Suppl. Figure 13 &  14). Further, Ingenuity pathway analysis (IPA) suggested that several inflammatory pathways including innate and adaptive immunity were upregulated in > 12 M TG rats compared with 3 M TG rats (Fig. 6b). Most interestingly, PD-1, PD-L1 cancer immunotherapy pathways, antioxidant action of vitamin C, RhoGDI signalling, Apelin cardiac fibroblast signalling pathway and LXR/RXR activation pathways were downregulated significantly in older TG (> 12 M) compared with 3 M TG rats (Fig. 6b, c). More specifically, with ageing NFAT regulation of the immune response, cardiac hypertrophy signalling, neuroinflammation signalling pathway, G-beta gamma signalling, ephrin receptor signalling and opioid signalling pathway were upregulated with ageing in > 12 M TG rats compared to 3 M TG rats (Fig. 6b-e). Additionally, whole genome wide analysis suggested that programmed cell death pathway genes were decreased, whilst immune system pathway genes were increased in old TG rats compared to young TG rats (Fig. 6b & Suppl. Figure 13). Therefore, we tested the adaptive immune cells of younger (3 M) and older (> 12 M) TG rats with their respective age matched WT controls. We found that ageing led to a significant increase in inflammatory IFN-γ cytokines from CD4^+^ T cells and also increased CD4^+^ T cells (Fig. 6f), whereas no significant difference was found in 3 M age between WT and TG (data not shown). Overall, data are suggesting that ageing TG rats have an activated inflammatory environment compared with younger TG rats.

### Antibiotic cocktail treatment affects the gut bacterial diversity, metabolite production and gene expression in the gut

**Fig. 7:**
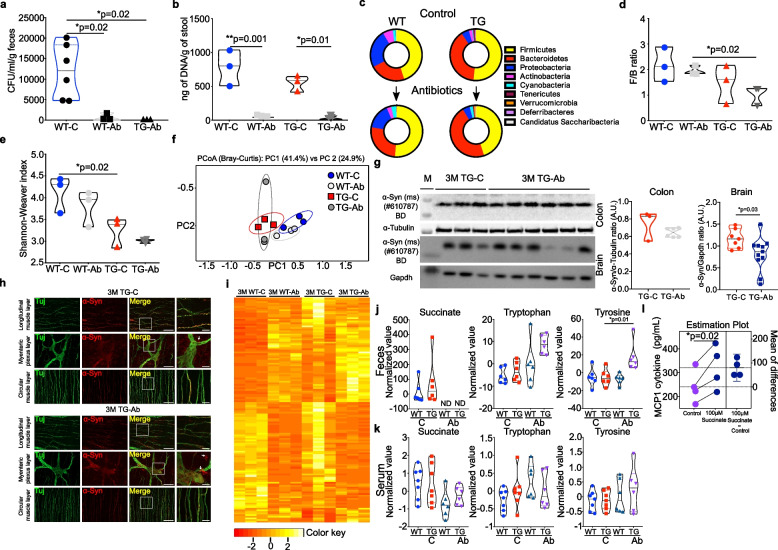
Treatment with broad-spectrum antibiotic cocktail leads to reduced gut microbiome load, α-Syn expression and succinate levels. **a** CFU measurement after broad-spectrum antibiotics treatment in WT and TG rats after equal amount of feces. **b** Amount of total DNA after antibiotic cocktail treatment in WT and TG rat fecal samples. **c** Antibiotic affected the bacterial phyla differentially for WT and TG rats. **d** F/B ratio was decreased after antibiotic treatment in WT and TG rats respectively, however, no significance difference was observed. **e** α-diversity measurement at genera level after antibiotics treatment for WT and TG rats. **f** β-diversity measurement (genera level) using Bray–Curtis method. Antibiotic treatments affect the clustering of intestinal bacteria. **g** Immunoblot image of α-Syn expression in ENS and olfactory bulb brain region from control and antibiotic treated TG rats and quantification of immunoblots are shown in violin plots. **h** Whole mount staining of MS tissue layer (longitudinal, circular and myenteric muscle layer) and expression of neuronal (Tuj) and α-Syn expression. **i** Heat-map presenting the RNA-seq data from control and antibiotics treated 3 M rats. **j**, **k** Succinate, tryptophan and tyrosine levels in the feces and serum from control and antibiotic treated WT and TG (3 M) rats. **l** Treatment of SH-SY5Y cells with succinate treatment (100 µM) significantly increased the MCP1 levels. *P* value significance represents *p ≤ 0.05

To understand whether reducing the gut bacterial load could lead to changes in the inflammatory environment or α-Syn expression, we treated the WT and TG rats at 2.5 M of age with broad-spectrum antibiotics for two weeks and found no change in body weight (Suppl. Figure 15). Antibiotic treatment led to a reduction of live bacterial load and further reduced the amount of the total DNA obtained from an equal weight of the stool samples (Fig. 7a, b). The 16S rRNA amplicon sequencing suggested a tendency to reduce the F/B ratio and bacterial α-diversity (phylum level) after antibiotic treatment for WT and TG rats respectively. However, no significant difference was observed (Fig. 7c-e). β-diversity (PCoA of taxonomy using Bray–Curtis at phylum level) data suggested that antibiotic treated rats clustered into different groups compared with untreated rats (Fig. 7f).

We isolated the *Tunica muscularis* containing the myenteric plexus and found by immunoblotting that antibiotic treatment did not affect the total α-Syn expression in the ENS. However, there was a significantly reduced α-Syn expression in the forebrain olfactory bulb (left) region only. (Fig. 7g). Yet, since there was a considerable interindividual difference of α-Syn in the olfactory bulb, it is up to future investigations to evaluate the biological relevance of this finding. Wholemount staining of the *Tunica muscularis* of TG rats from antibiotic treatment and control groups revealed that the expression of α-Syn was present in the myenteric plexus as well as in nerve fibers in the circular and longitudinal muscle layers and that there was no change in expression in response to the antibiotic treatment (Fig. 7h).

Moreover, we performed the RNA-seq analysis of the colon MS tissue layer, we found that several genes were differentially regulated after antibiotic treatment for WT and TG rats respectively (Fig. 7i). IPA analysis suggested that the antibiotic treatment affected 478 genes in TG rats and 521 genes in WT rats (normalised for both the genotypes independently TG-Ab/TG-C and WT-Ab/WT-C). Out of these genes 165 were common in WT and TG. These changes could be caused either by the reduced gut microbiome load or the direct effects of the applied antibiotic (Suppl. Figure 16). Interestingly, *Dnase1* and *Trpv1* were two of the genes that were upregulated after antibiotic treatment in TG rats. Genes that were upregulated in both WT and TG rats after antibiotic treatments included *Hdac-5*, *CD86* and *Pink1*, whereas *RoRc*, *Spink4*, *Hsp90b1* were downregulated (Suppl. Figure 16).

Furthermore, we performed ^1^H-NMR metabolomics of antibiotic treated rats and identified 45 fecal and 38 serum metabolites. We found that antibiotic treatment was followed by profound effect on the fecal SCFAs, amino acids (e.g. tyrosine, tryptophan, lysine was reduced in WT rats after antibiotics, whereas no change was observed in TG rats) and sugars (Suppl. Figure 17a & b) while for serum only slight changes could be observed (Suppl. Figure 17c & d). Most strikingly, multivariate VIP score analysis of serum samples (Suppl. Figure 17e) identified most strikingly reduced succinate levels (Suppl. Figure 17f) alongside decreased occurrence of the ketone body 3-Hydroxybutyrate (Suppl. Figure 17g) in both WT and TG rats after antibiotic treatment. Tyrosine was significantly increased with ageing in TG rat feces after antibiotic treatment, while Tryptophan also had a similar trend but not reaching statistical significance (Fig. 7j). Succinate was totally absent after antibiotic treatment in the feces, thus, suggesting the potential role of succinate production by bacterial metabolism (Fig. 7j). These data signify that microbiome manipulation affects the bacterial load, gene expression and metabolite production.

### Succinate creates a conducive environment for chemoattraction & inflammation

To understand the importance of succinate in neuroinflammation, we used SH-SY5Y neuroblastoma cells. We treated the cells with varied concentrations of succinate (50, 100, 200, 250, and 500 µM) for 48 h and collected the supernatant. Supernatants were subjected to 13-plex cytokine analysis and found that macrophage chemoattractant protein 1 (MCP1) was significantly upregulated at 100 µM concentrations compared with the control samples (Fig. 7k and Suppl. Figure 18a). Presence of succinate receptor 1 (SUCNR1 or GPR91) expression was confirmed in SH-SY5Y neuroblastoma cells by immunofluorescence (Suppl. Figure 18b). Furthermore, we performed proteomic analysis from control and 100 µM treated cells and found that several proteins were significantly changed (Suppl. Figure 18b). Based on in silico based Metascape pathway analysis [58], we found that succinate treatment could decrease cellular response to stress, regulation of protein catabolic pathways and brain-derived neurotrophic factor (BDNF) signalling pathway proteins. Whilst, succinate treatment could help to reduce the cytosolic Ca^2+^ levels and increase brain development pathway proteins, VEGF-VEGFR2 signalling and IL-12 inflammatory pathway proteins (Suppl. Figure 16c-d). Overall, succinate treatment results in increase in chemoattraction, dysregulated inflammatory pathway, with cellular stress and calcium probably creating an environment for dysfunction neuronal signalling in vitro conditions.

## Discussion

Recent emerging reports have investigated gut microbiome dysregulation and its role in the pathogenesis of PD [1, 19, 20, 33, 59]. Most of the studies propose that PD patients undergo changes in the relative quantity of particular types of bacterial genera, and due to these changes in bacterial load, the metabolism of PD patients is affected [20, 60]. Altered bacterial genera/species and their changes to metabolite production may also inadvertently modulate the immune response, ultimately leading to inflammation in various tissues and cellular components of the gut including enteric neurons, glial cells, and immune cells [35]. However, the data from PD patients are always limited in numbers and often have a high variance in terms of age differences, body mass index, exposure to antibiotic, diet, ethnicity etc. To study and understand how the gut microbiome is involved in PD pathology, the above-mentioned factors need to be controlled to have a clear understanding. Moreover, it would be critical to identify patients in the pre-clinical stage who are not experiencing any motor and non-motor symptoms. To address these issues, rodent models (mouse or rats) are useful for identifying and performing functional and mechanistic research on host-microbiome interactions as they allow manipulation of genome, environment, and gut microbiome composition [61]. In this study, using the TG PD rat model, we observed that overexpression of α-Syn modifies the gut microbiome, metabolite production and induces inflammation and short-term manipulation of the gut microbiome using antibiotic cocktail decrease the forebrain α-Syn expression.

Current findings have demonstrated that the intestinal microbiota interacts with the autonomic and central nervous system via diverse pathways, including the ENS and Vagal nerve [35]. Studies in PD patients have described that microbial composition in feces and mucosa were significantly different at multiple levels [1, 19, 59]. The diversity of fecal bacterial communities was not largely different between PD and healthy control subjects [50]. Significant differences in α-diversity between PD and healthy control groups were primarily observed at the phylum level and PD disease duration correlated with the majority of taxa, e.g., Bacteroidetes and Proteobacteria being positively correlated and Firmicutes negatively correlated [1]. Additionally, few patient studies reported that Prevotellaceae family or *Prevotella* genus was less abundant in the PD patients [19, 20]. We found that *Prevotella* bacterial levels were higher in younger TG rats compared with WT and that at an older age, bacterial levels were lower in TG PD rats, which is consistent with PD patient studies [22].

Furthermore, our current findings demonstrate that α-Syn expression leads to change in gut microbiota composition and more specifically the *Lactobacillus* and *Alistipes* genera in early young stage, non-symptomatic younger age *versus* older aged, symptomatic TG rats. Additionally, gender difference could also be involved in shaping the abundance of *Lactobacillus* and *Alistipes* genera. Abundance of *Lactobacillus* genera is in contrast [19, 24–28] with many PD patient studies, whilst some are advocating in favour of our study [20, 30, 31].The conundrum between murine models and microbiome of PD patients is indeed a great concern to draw a concrete conclusion. A recent metagenome-based of PD patients study revealed higher levels of *L. fermentum, L. gasseri, L. paragasseri, L. reuteri, L. rhamnosus, L. salivarius* despite healthy controls administered a diet rich in probiotics [29]. However, when we examined our metagenomics data from PD rat model, we found that *L. reuteri, L. johnsonii, L. murinus* were significantly lower in PD rats compared with control group. Only one *L. reuteri* species was common among PD patients and TG PD rats. This species was least abundant in PD patients among all other *Lactobacillus* species. Another probiotics genus, *Bifidobacterium* abundance among human patients and murine models were discordant. To explain this difference in findings, it may be possible that the animal models do not fully recapitulate what is occurring in the human microbiome caused by α-Syn over-expression. Perhaps α-Syn over-expression followed by aggregation with ageing could induce inflammation [11] and potentially target the microbiome abundance among species. Moreover, the multi-factorial nature of human PD creates a different intestinal environment than α-Syn over-expression alone in the PD TG rat model. Interestingly, in naïve de novo PD patients from the Netherlands and Finland had no change in *Lactobacillus* compared with control groups [32]. Another study suggested that PD patients have increase of opportunistic pathogens which is independent of levodopa and an increase in abundance of probiotics *Lactobacillus* and *Bifidobacterium* were correlated with increasing dose of levodopa [62]. A few murine PD models (α-Syn and rotenone) also suggested that decrease in *Lactobacillus* abundance in cecal content, thus, highlighting the validity of the data presented in this study [44, 63]. Nonetheless, the key aspect that the *Alistipes* genus was higher in PD patients and murine PD models. Some of the findings are reproducible in human patients and murine PD models. Thus, it is plausible that different genera and species could have different outcomes on PD pathology is subject to furthur research.

*Alistipes* abundance was increased with ageing in TG rats suggesting an increase in inflammation in the colon. Similarly, like the rat PD model, *Alistipes* was reported to be increased in PD patients [31]. Reports in other model system such as colon carcinoma and inflammatory bowel disease suggested that increased abundance of *Alistipes* could lead to enhanced inflammatory phenotype in these models [64–66]. It appears that *Alistipes* could be a slow inducer of inflammation in the colon with ageing TG rats, however, further study is warranted. Moreover, our metabolites and immunophenotyping data highlighted enhanced inflammation comes with ageing in TG rats. *Lactobacillus species* such as *L. reuteri, L. casei, L. plantarum, and L. fermentum and strains such as L. mucosae AN1 and L. fermentum SNR1* are considered anti-inflammatory [67, 68], subsequently there could be a possibility that a low abundance of this bacteria allows a niche for the development of more opportunistic inflammatory bacteria such *Alistipes* and *Desulfovibrio* genera. Additionally, α-Syn overexpression could be key in governing the abundance of *Lactobacillus* in homozygous TG rats. Our maternal microbiome study could explain a decreased abundance of *Lactobacillus* as heterozygous mothers have an equivalent percentage of WT, while homozygous TG rats have less abundant *Lactobacillus*. Thus, the present data are implying that genetics is a major driving force in shaping host-microbiome interactions in our PD rat model. Further, in vivo validation with the individual gut bacterial genera/strain is required to support this notion.

A major PD patho-histological hallmark is the occurrence of eosinophilic cytoplasmic neuronal inclusions (Lewy bodies) as well as α-Syn positive neuronal processes called Lewy neurites [4, 17, 69]. Immunohistochemical data from PD patients before the onset of disease (2–5 years) suggested that α-Syn positive neurons were present in sigmoid colon mucosa [17]. Thus, as suggested earlier, it is possible that inflammation-induced oxidative stress could lead to misfolding of α-Syn in TG rats, and successively α-Syn pathology spreading to the brain in a prion-like fashion [3, 12, 13, 15, 17, 70]. It is also conceivable that changes in intestinal bacteria over the course of ageing lead to an increase in intestinal endotoxins, which could enhance the local or systemic inflammation and induce oxidative stress leading to disruption of intestinal barriers or enteric neuroinflammation [34, 71]. In line with this, we found that ageing increased gut and systemic inflammation in TG rats and several metabolites such as succinate, lactate, glutamate and 4-Hydroxyphenylacetate in the blood serum and feces of aged TG rats (a systemic route of inflammation). Our correlation analysis revealed that the *Alistipes* genus could be involved in succinate production. It was recently reported that succinate was highly abundant in urine from PD patients compared with healthy controls, furthermore, succinate was correlated with motor score, constipation [53] and hence disease severity [72]. Thus, succinate could be used as a prognostic marker for PD pathology. Additionally, the blood serum levels of other anti-inflammatory metabolites such as phenylacetate and tyrosine/tryptophan were decreased in ageing TG rats. Our data suggest that with ageing, an inflammatory environment is indeed to be prominent. The inflammatory niche and dysregulated bacterial composition may accelerate the accumulation or aggregation of phosphorylated α-Syn in the myenteric plexuses of TG rats. This is in keeping with previously described results in the brain which suggested that aged TG rats had a higher accumulation of α-Syn [3, 40].

Several chronic autoimmune intestinal diseases such as inflammatory bowel disease, celiac disease, type 1 diabetes, multiple sclerosis, and systemic lupus erythematosus are linked with increased intestinal permeability also referred to as “leaky gut” [73]. Gut leakiness in patients with a genetic susceptibility to PD may be a pivotal early step promoting a pro-inflammatory/oxidative environment contributing to the initiation and/or progression of the PD process [34]. In line with this, our ‘Ussing chamber’ data highlighted that ageing TG rats tended to have significantly less salt entry into intestinal cells than age-matched WT control rats. Intestinal gut permeability was increased with ageing in both TG and WT rats compared with young rats of both genotypes. Thus, we postulate that a lower uptake to Na^+^ ions from the stool could have deleterious effects on *Lactobacillus.* This agrees with a recent study, as increased NaCl intake reduced the *Lactobacillus* in mice model [74]. Furthermore, serum sodium levels are inversely associated with dyskinesia in PD patients as lower levels of serum sodium are more likely to have dyskinesia [75]. Nevertheless, further understanding of the physiology of ion channels and the gut microbiome would be helpful to understand gut-related onset of PD pathology.

Recently several inflammatory proteins such as calprotectin were found to be upregulated in the feces of PD patients [56, 57]. Other inflammatory proteins such MCP1 also correlated with PD progression [76] as well as in the PD susceptible murine model DJ-1 [37]. Our recent data in the PD murine model suggested that several inflammatory proteins were also be present in the feces of TG rats [44]. Similarly, we also found that in the rat model ageing increases levels of calprotectin levels in the feces and serum. Our RNA-seq data pointed that several inflammatory genes such as NFAT regulation of immune response, neuroinflammation and Th1 immune response pathways were upregulated with ageing and reduction in PD-1, PD-L-1 immunomodulatory pathways, antioxidant pathways and LXR/RXR activation in TG rats. Further, these data were validated using immunophenotyping and found that activated CD4^+^ T cells had increased expression of IFN-γ in TG rats. IFN-γ producing T cells are involved in the induction of inflammation in the brain and activate the inflammatory microglial cells in the *SNpc* region, thus, leading to neuronal cell death and nitrated α-Syn [77]. Most importantly PD-1 inhibitors are involved in neurological toxicities in patient with non-small-cell lung cancers [78] as well as melanoma patient [79]. Thus, reduction in PD-L-1 pathways could also participate in neurodegeneration including PD as our RNA-seq data from colon tissues suggest that PD-1, PD-L-1 immunomodulatory pathways were drastically reduced in TG rats. However, further validation studies are warranted to understand these pathways in PD pathology.

Our microbiome manipulation study by use of broad-spectrum antibiotic again highlights the importance of the gut microbiome and, in particular its ability to modify metabolite production which in theory could be directly involved in the gut inflammation and, thus PD pathology. The antibiotic cocktail treatment completely abolished the SCFAs, reduced the succinate production, and increased the production of the amino acids; tyrosine/tryptophan. Tyrosine is the precursor of dopamine synthesis and tryptophan in serotonin production respectively [80]. Accordingly, it could be conceivable that gut bacteria could be regulating the succinate, tyrosine and tryptophan production and could influence neurodegenerative processes by regulating dopamine and serotonin production. Furthermore, it could also be plausible that the gut microbiome manipulation by short-term antibiotic cocktail treatment could help to modify metabolite production due to dysregulated gut microbiome in PD patients which might improve PD symptoms. Succinate is a pro-inflammatory intermediatory metabolite of tri-carboxylic acid cycle and involved in activation of macrophages and hypoxia induction [81]. Succinate treatment could be implicated in recruitment of inflammation by activating neuronal and immune cells as implicated in this study. However, a recent study on 1-Methyl-4-phenyl-1,2,3,6-tetrahydropyridine (MPTP) mouse of PD suggested succinate treatment could reduce dopaminergic neuronal loss, a sharp contrast to our data [82]. However, further detailed investigations are required before it could be implemented for PD patients.

## Conclusion

In summary, overall, the studied TG rat model overexpressing α-Syn presented changes in the gut microbiota, intestinal inflammation, dysregulated metabolites in the feces/plasma, α-Syn aggregation in parallel with ageing. Some of these characteristics can similarly be detected in human PD patients. Microbiome alteration using short-term antibiotic cocktail is able to reduce the metabolite production in the feces/plasma and α-Syn expression in the forebrain of TG rats. We previously showed that this model exhibits early alterations in avoidance and novelty-seeking behaviour and late motor decline [40] where most of the bacterial diversity and metabolites are drastically changed. Our studies showed that α-Syn aggregation could be detected within the walls of the colon of TG rats before the brain pathology develops. Based on this study, we could suggest that understanding the microbiome/metabolite dynamics together with physiology changes of the gut could provide a clue to Parkinson’s disease even at the prodromal stage.

## Supplementary Information

**Additional file 1: Suppl. Fig. 1. **The gut microbiome dynamics with ageing, decreased *Lactobacillus* and increased *Alistipes* bacterial genera in the TG rats. **a** 16S rRNAgene amplicon sequencing was performed on samples from 1, 2, 2.5, 3, 6 and 14 M of age group. Total number of sequences read obtained from WT and TG samples were similar and a significant difference was not observed. **b** The α-diversity within each group was estimated using Shannon-Weaver index based on phylum level classifications. No significant difference was noticed between WT and TG rats at any age group. Data are representedin Box and Whisker plots. **c** Age dynamics of phyla Firmicutes, Bacteroidetes, Actinobacteria, Proteobacteria and Verrucomicrobia. Each phylum was significantly different at a particular age as shown in the figure with significant level. **d** The Shannon-Weaver index and Chao1 at genus level at a particular age. Significant change is shown in asterisk for a defined age group. **e** The dynamic representation of *Prevotella, Alloprevotella, Parabacteroides, Alistipes Lactobacillus, Turicibacter, Ruminococcus, Desulfovibrio *and *Bifidobacterium *with ageing in WT and TG respectively. A significant difference is shown for a particular age group. *P* value significance represents *p≤0.05, **p≤0.01 and ***p≤0.001. **Suppl. Fig. 2. **Clustering of bacterial phyla for different age group. Bacterial clustering figure showed change in the bacterial abundance with ageing. **Suppl. Fig. 3. **Genetic makeup of animal could be an important factor for microbiome composition development. **a** A study was carried out from 4M old heterozygous females 6; 2 females/cage; colour coded) were kept in 3 different cages and when females were pregnant then male were separated. Fecal samples were collected from the females after pups were born. After 3 weeks of age, pups were separated from females and genotyped and kept in WT and homozygous TG groups in 5 different cages as explained in the Fig. Fecal samples were sequenced by 16S rRNA sequencing and analysed by MEGAN-CE software. Clustering analysis at species level suggested that WT and homozygous TG rats’ clusters differentially with each other as well as with mother’s microbiome. **b** The percentage abundance of *Lactobacillus *genus and species in two different facilities at the same age group intwo different cohorts. *P* value significancerepresents *p≤0.05. **Suppl. Fig. 4. **Similar presence of *Lactobacillus *genus in two different facilities. **a** At 3M of  age species comparison study showed that two different facilities had a similar abundance of *Lactobacillus *species. **b** The percentage abundance of *Lactobacillus *genus and species in two different facilities at the same age group in two different cohorts. **Suppl. Fig. 5. **Coprophagy is one of route and method for the microbial manipulation in PD rat models. **a** Schematic diagram for the bacterial manipulation by cage transplant. **b** Total no of sequence reads. **c** Hollow pie charts represent the bacterial composition at phylum level before and after fecal transplant. **d** F/B ratio before and after faecal transplant. **e** Pie charts show the bacterial composition before and after faecal transplant in WT and TG PD rat model. **f** Bacterial composition in individual WT and TG rats before and after transplant and represented as a heat-map. **g** Microbiome abundance in WT and TG rats from co-housed rats since childhood together in the same cages and N=7 TG). **Suppl. Fig. 6. **Shotgun sequencing based confirmation of microbiome diversity and abundance. The gut microbiome dynamics with ageing, decreased *Lactobacillus *and increased *Alistipes *bacterial genera in TG rats. **a** Total no of sequence reads after shotgun sequencing obtained from WT an TG samples were similar. **b** Rarefaction plots for all samples. **c** Clustering of bacterial at species levels from WT and TG rats. **d** Significantly changed bacterial species in WT and TG rats. **e, f **The genomic metabolic profiles of > 12 M feces microbiome based on shotgun sequencing. **Suppl. Fig. 7. **The metabolic profiles of **a** 3M feces and **c** serum show no clear separation of WT and TG rats in the PLS-DA scores plots and also heat-maps show no clear clusters. By contrast, after >12M TG and WT samples could be clearly separated in the PLS-DA scores plots in both** b**  feces and **d** serum analysis. The most prominent metabolites in the VIP score analysis for feces samples were succinate and glutamate. For serum, 3M rats showed high glucose levels in TG and high lactate levels in WT, while at >12M TG rats showed highly increased lactate and succinate levels. **Suppl. Fig. 8. **The serum metabolome analysis of WT rats **a** revealed significant metabolite fold changes during ageing. At >12M of life time, WT serum were composed with increased levels of formate, isoleucine, valine, taurine, creatine, glutamine and lysine and decreased values of betaine, N,N-dimethylglycine, glutamate, sn-glycero-3-phosphocholine, citrate and 3-hydroxybutyrate. Succinate was increased at 3 M of age, however, above the significance threshold. Results from the same spectra with multivariate PLS-DA VIP scores analysis **c** provided similar results for WT while TG rats showed mainly an in increase in lactate and succinate. A comparison of the feces metabolome during aging showed both for WT and TG adecrease in succinate levels **b** for which a correlation analysis was performed. **Suppl. Fig. 9. **Correlation between microbiome abundance and metabolites **a** > 12 M fecal microbiome and metabolite **b** > 12 M microbiome abundance and serum metabolites. **c** Fecal microbiome and fecal metabolites significance levels using Pearson correlation coefficient. **Suppl. Fig. 10. **Fragmented α-Syn and expression in ENS from the colon of >12M TG rats. **a **Estimation of fragmentation in α-Syn in from the colon ENS. Two major truncated fragments were found in ENS as shown in α-Syn immunoblot image. **b** Expression of α-Syn in the colon muscular layer. **Suppl. Fig. 11.** Accumulation of α-Syn in TG rat colon. **a** Colon tissues were digested with trypsin or Proteinase K for 2 hours and performed with human specific antibodies as described in materials and methods section. IHC showed the accumulation of human/rat specific total α-Syn in both 2M and >12M TG rats, whereas in WT α-Syn was not detected. After digestion aggregated α-Syn was retained in TG rats suggested the accumulation. **b** Detection of aggregated phosphorylated α-Syn in the colon tissues in young and old TG rats after trypsin digestion. **c** Proteinase K digestion of the colon to investigate an accumulation of aggregated total α-Syn protein in TG rats. **Suppl. Fig. 12. **Human α-Syn is truncated in TG rats. **a** The expression of endogenous α-Syn in rat colon and upto 4 different truncated α-Syn can be observed in the colon of the TG rats, but the appearance of these different bands differs between individual and does not appear to be correlated with age. In some TG rats, the expression of α-Syn is similar than in the WT rats but showing truncated fragments of lower molecular weight. No pathologic phosphorylation could be detected by immunoblotting method. **b** DSP treatment increases α-Syn immunodetection in WB. The detection of the truncated α-Syn band is not dependent on boiling the samples, as similar band is also present in non-boiled samples, but its signal is increased in presence of the crosslinker DSP. **c** Using different α-Syn antibodies covering different epitopes of the sequence were used to detect the fragmentation of α-Syn. N-terminal antibodies did not detect the first truncated fragment present in sample 1 and 2- Detected the second truncated fragment present in sample 2 and 3. Detected an extra truncated fragment insample 1 with a similar molecular weight the second truncated fragment. C-terminal antibodies do not detect the first and second truncated fragment present in sample 1 and 2, but only upto residue 125. SA3400  does not detect these truncated forms. The extra truncated fragment in sample 1 is detected by all c-terminal antibodies. 1st truncated band is observed with α-Syn 211, 14H2L1, LB509 and FL140, however it was not observed with SA3400, EP1466Y, N19  and Ab6176. The fact that the 1^st^ truncated band was not observed by SA3400, however it was observed by all the other C-term antibodies upto 125, suggest that it might be a N-terminal fragment with the truncation located between the position 125 and 131. Further, the truncated fragment was not observed by any of the three N-term antibodies tested, thus, further suggesting that the fragment was also truncated at N-terminal part. The 2^nd^ band was observed with N19, Ab6176, EP1646Y, 14H2L1and LB509 and not observed with SA3400. Thus, detected band was observed by all the N-term antibodies but not by the most distant C-term antibody suggesting that it was truncated in the C-terminal part, probably around the position 120. **d** An illustration is showing possible aberrant alternative splicing variant sites. **Suppl. Fig. 13.** RNA-seq analysis from 3M and >12M old WT and TG colon epithelial tissues. **a** Differentially expressed genes at 3M age in WT and TG rats. Total no of differentially expressed genesbased on absolute log fold-change of at least 1, whereas at >12M of age total no of differentially expressed were 1808 based on absolute log fold-change of at least 1 . There was no statistical significance based on false discovery rate  for 3M, however at >12M 264 genes were different. **b** Clustering of >12M WT and TG epithelial samples. **c, d** IPA analysis of adaptive  and innate  for the >12M samples. **e** Different inflammatory gene expressions. **Suppl. Fig. 14. **Reactome transcriptome wide overview analysis of **a** 3M and  **b** >12M epithelial RNA-seq samples. The figure shows a genome-wide overview of the results of pathway analysis 3M and>12M. Reactomepathways are arranged in a hierarchy. The center of each of the circular “bursts”is the root of the one top-level pathway. Each step away from the center presents the next level lower in the pathway hierarchy. The colour code denotes over representation of a particular pathway in the input dataset. Light grey signifies pathways which are not significantly over-presented. At 3M programmed cell death pathway genes were over presented whereas at >12 M programmed cell death pathway gene were downregulated. Further, at >12M M there were an over-representation of immune system genes and less representation of programmed cell death genes. **Suppl. Fig. 15. **Antibiotic treatment strategy and health status of the rats. **a** Antibiotic treatment plan for young rats. **b** Antibiotic treated health based on body weight. **Suppl. Fig. 16. **Antibiotic treatment increase TRPV1 and Dnase1 in the colon. **a** Clustering of the samples in the control and antibiotic treated groups. **b** Genotype analysis in control and antibiotic treated colon samples. Total 165 genes were found to be commonly regulated by antibiotic. More differential gene expression in WT compared with TG rats. Expression of TRPV1, Dnase1 in WT and TG colon. **Suppl. Fig. 17. **Metabolite changes in the feces and serum after antibiotics treatment at 3M age. **a** Antibiotic treatment results in a clear separation of feces samples in the PLS-DA analysis and **b** heat map. **c** While the effect on the corresponding serum scores plots and **d** heat-map is rather small. **e, f, g** Multivariate VIP score analysis identified decreased succinate in both TG and WT control samples after antibiotic treatment alongside the ketone body 3-Hydroxybutyrate. Box and whisker plots showed the succinate and 3-Hydroxybutyrate in control and antibiotic treated samples. **Suppl. Fig. 18. **Succinate treatment increase the chemokine & inflammatory  pathway proteins in neuroblastoma cells. **a** SH-SY5Y neuroblastoma cells were treated with different concentrations succinate as mentioned. Supernatants were collected from each conditionand subjected to 13-plex cytokine analysis. MCP1 was significantly increased after 100 μM succinate treatment. Paired Student’s t-test was applied for statistical analysis. *P* value significance represents *p≤0.05. **b** GPR91 or SUCNR1 expression in SH-SY5Y neuroblastoma cells . **c** Volcano plots for differentially regulated proteins. **d** Pathway analysis for downregulated **e** upregulated expressed proteins.

## Data Availability

We have provided most of the data with pre-print severe https://www.biorxiv.org/content/10.1101/2020.12.23.424226v. Metagenomics shotgun sequencing and RNA transcriptomics sequencing data are available under IDs:PRJNA986826 and GEO accession:GSE236012 respectively. The datasets used and/or analysed during the current study are available from the corresponding authors on a reasonable request.

## Abbreviations

PD: Parkinson’s disease
DA: Dopaminergic
LBs: Lewy bodies
LNs: Lewy neurites
SNpc: *Substantia nigra pars compacta*
α-Syn: α-Synuclein
GIT: Gastrointestinal tract
EEC: Enteroendocrine cells
TG: α-Synuclein Transgenic mice
GF: Germ-free
^1^H-NMR: Proton nuclear magnetic resonance spectroscopy
IPA: Ingenuity Pathways Analysis
SCFAs: Short-chain fatty acids
ELISA: Enzyme linked immunosorbent assay
IHC: Immunohistochemistry
F/B: Firmicutes/Bacteroidetes ratio
