# Supplementary material for: Overexpression of human alpha-Synuclein leads to dysregulated microbiome/metabolites with ageing in a rat model of Parkinson disease
Source: Mol Neurodegener. 2023 Jul 4;18:44. doi: 10.1186/s13024-023-00628-1 (PMC10318726; doi:10.1186/s13024-023-00628-1)
Supplement: Supplementary file 1 — Additional file 1: Suppl. Fig. 1. The gut microbiome dynamics with ageing, decreased Lactobacillus and increased Alistipes bacterial genera in the TG rats. a 16S rRNAgene amplicon sequencing was performed on samples from 1, 2, 2.5, 3, 6 and 14 M of age group. Total number of sequences read obtained from WT and TG samples were similar and a significant difference was not observed. b The α-diversity within each group was estimated using Shannon-Weaver index based on phylum level classifications. No significant difference was noticed between WT and TG rats at any age group. Data are representedin Box and Whisker plots. c Age dynamics of phyla Firmicutes, Bacteroidetes, Actinobacteria, Proteobacteria and Verrucomicrobia. Each phylum was significantly different at a particular age as shown in the figure with significant level. d The Shannon-Weaver index and Chao1 at genus level at a particular age. Significant change is shown in asterisk for a defined age group. e The dynamic representation of Prevotella, Alloprevotella, Parabacteroides, Alistipes Lactobacillus, Turicibacter, Ruminococcus, Desulfovibrio and Bifidobacterium with ageing in WT and TG respectively. A significant difference is shown for a particular age group. P value significance represents *p≤0.05, **p≤0.01 and ***p≤0.001. Suppl. Fig. 2. Clustering of bacterial phyla for different age group. Bacterial clustering figure showed change in the bacterial abundance with ageing. Suppl. Fig. 3. Genetic makeup of animal could be an important factor for microbiome composition development. a A study was carried out from 4M old heterozygous females 6; 2 females/cage; colour coded) were kept in 3 different cages and when females were pregnant then male were separated. Fecal samples were collected from the females after pups were born. After 3 weeks of age, pups were separated from females and genotyped and kept in WT and homozygous TG groups in 5 different cages as explained in the Fig. Fecal samples were sequenced by 1 [file 13024_2023_628_MOESM1_ESM.pdf]

Overexpression of human alpha-Synuclein leads to dysregulated microbiome/metabolites with ageing in a rat model of Parkinson disease

Yogesh Singh[1,2]*, Christoph Trautwein[3], Joan Romani[4], Madhuri S Salker[5], Peter H Neckel[6], Isabel Fraccaroli[1], Mahkameh Abeditashi[1], Nils Woerner[1], Jakob Admard[1,2], Achal Dhariwal[7], Morten KD Dueholm[8], Karl-Herbert Schäfer[9], Florian Lang[10], Daniel Otzen[11], Hilal A Lashuel[4], Olaf Riess[1,2]* and Nicolas Casadei[1,2]*

[1]Institute of Medical Genetics and Applied Genomics, University of Tübingen, Calwerstaße 7, 72076, Germany

[2]NGS Competence Centre Tübingen (NCCT), University of Tübingen, Calwerstaße 7, 72076, Germany

[3]Werner Siemens Imaging Centre (WSIC), Department of Preclinical Imaging and Radiopharmacy, University of Tübingen, Röntgenweg 13, 72076, Germany

[4]École polytechnique fédérale de Lausanne (EPFL) BMI SV LMNN Station 19, 1015 CH Lausanne, Switzerland

[5]Research Institute of Women's Health, University of Tübingen, Calwerstaße 7/6, 72076, Germany

[6]Institute of Clinical Anatomy and Cell Analysis, University of Tübingen, Österbergstraße 3, 72074, Germany

[7]Institute of Oral Biology, University of Oslo, Sognsvannsveien 10, 0316, Norway

[8]Department of Chemistry and Bioscience, Aalborg University, Fredrik Bajers Vej 7H, 9220 Aalborg, Denmark

[9]Enteric Nervous System working Group, University of Applied Sciences Kaiserslautern, Zweibrücken Campus, Amerikastrasse 1, 66482 Zweibrücken, Germany

[10]Institute of Vegetative Physiology, University of Tübingen, Wilhelmstaße 56, 72074, Germany

[11]Interdisciplinary Naonscience Center (iNANO), Aarhus University, Gustav Wieds Vej 14, 8000, Aarhus, Denmark

Short Title: Microbiome dynamics in PD pathogenesis

Key words: Gut microbiome, PD, intestinal permeability, α-Synuclein, antibiotics

*Address for Correspondence:

Dr Yogesh Singh
Institute of Medical Genetics and Applied Genomics,
University of Tübingen,
Calwerstaße 7, 72076, Germany
Phone: +49 7071 29 78264
Fax: +49 7071 29 25355
Email: yogesh.singh@med.uni-tuebingen.de

Suppl. Figure legends

Suppl. Fig. 1 The gut microbiome dynamics with ageing, decreased *Lactobacillus* and increased *Alistipes* bacterial genera in the TG rats. (a) 16S rRNA gene amplicon sequencing was performed on samples from 1, 2, 2.5, 3, 6 and 14 M age group WT and TG respectively. Total number of sequences read obtained from WT and TG samples were similar and a significant difference was not observed. (b) The alpha diversity within each group (WT and TG rat) was estimated using Shannon-Weaver index based on phylum level classifications. No significant difference was noticed in between WT and TG rats at any age group. Data are represented in Box and Whisker plots. (c) Age dynamics of phyla Firmicutes, Bacteroidetes, Actinobacteria, Proteobacteria and Verrucomicrobia. Each phylum was significantly different at particular age as shown in the figure with significant level. (d) The Shannon-Weaver index and Chao 1 (alpha diversity) at genus level at particular age. Significant change is shown in asterisk for a defined age group. (e) The dynamic representation of *Prevotella, Alloprevotella, Parabacteroides, Alistipes Lactobacillus, Turicibacter, Ruminococcus, Desulfovibrio* and *Bifidobacterium* with ageing in WT and TG respectively. A significant difference is shown for a particular age group. P value significance represents *$p \leq 0.05$, **$p \leq 0.01$ and ***$p \leq 0.001$.

Suppl. Fig. 2 Clustering of bacterial phyla for different age group WT and TG rats. Bacterial clustering figure showed change in the bacterial abundance with ageing.

Suppl. Fig. 3 Genetic makeup of animal could be an important factor for microbiome composition development. (a) A study was carried out from 4M old heterozygous females (total no of female (F) 6; 2 females/cage (C); colour coded) were kept in 3 different cages and when females were pregnant then male were separated. Faecal samples were collected from the females after pups were born. After 3 weeks of age, pups were separated from females and genotyped and kept in WT and homozygous TG groups in 5 different cages (C1-5) as explained in the fig. Faecal samples were sequenced by 16S rRNA sequencing and analysed by MEGAN-CE software. Clustering analysis at species level suggested that WT and homozygous TG rats' clusters differently with each other as well as with mother's (Heterozygous) microbiome. (b) The percentage abundance of *Lactobacillus* genus and species in two different facilities at the same age group in two different cohorts. P value significance represents *$p \leq 0.05$.

Suppl. Fig. 4 Similar presence of *Lactobacillus* genus in two different facilities. (a) At 3M age species comparison study showed that two different facilities had similar abundance of *Lactobacillus* species. (b) The percentage abundance of *Lactobacillus* genus and species in two different facilities at the same age group in two different cohorts.

Suppl. Fig. 5 Coprophagy is one of route and method for microbial manipulation in PD rat models (a) Schematic diagram for bacterial manipulation by cage transplant. (b) Total no of sequence reads. (c) Hollow pie charts represent the bacterial composition at phylum level before and after faecal transplant. (d) F/B ratio before and after faecal transplant. (e) Pie charts show the bacterial composition before and after faecal transplant in WT and TG PD rat model. (f) Bacterial composition in individual WT and TG rats before and after transplant and represented as heat-map. (g) Microbiome abundance in WT and TG rats from co-housed rats since childhood together in the same cages (N=6 WT (n=3 male and n=3 female) and N=7 TG (n=4 male and n=3 female))

Suppl. Fig. 6 Shotgun sequencing based confirmation of microbiome diversity and abundance. The gut microbiome dynamics with ageing, decreased *Lactobacillus* and increased *Alistipes* bacterial genera in TG rats. (a) Total no of sequence reads after shotgun sequencing obtained from WT an TG samples were similar. (b) Rarefaction plots for all samples. (c) Clustering of bacterial at species level from WT and TG rats (>12M). (d) Significantly changed bacterial species in WT and TG rats. (e, f) The metabolic profiles of 14M feces microbiome based on shotgun sequencing.

Suppl. Fig. 7 The metabolic profiles of 3M feces (a) and serum (c) show no clear separation of WT and TG rats in the PLS-DA scores plots and also heat maps show no clear clusters. By contrast, after >12M TG and WT samples could be clearly separated in the PLS-DA scores plots in both feces (b) and serum (d) analysis. The most prominent metabolites in the VIP scores analysis for feces samples were succinate (high in TG for 3M) and glutamate (high in WT for >12M). For serum, 3M rats showed high glucose levels in TG and high lactate in WT, while at >12M TG rats showed highly increased lactate and succinate levels.

Suppl. Fig. 8 The serum metabolome analysis of WT rats (a) revealed significant ($p < 0.05$) metabolite fold changes ($FC > 1.2$) during ageing. At >12M of lifetime, WT serum were composed with increased levels of formate, isoleucine, valine, taurine, creatine, glutamine and lysine and decreased values of betaine, N,N-dimethylglycine, glutamate, sn-glycero-3-phosphocholine, citrate and 3-hydroxybutyrate. Succinate was increased at 3 months of age, however above the significance threshold. Results from the same spectra with multivariate PLS-DA VIP scores analysis (c) provided similar results for WT while TG rats showed mainly an in increase in lactate and succinate. A comparison of the feces metabolome during aging showed both for WT and TG a decrease in succinate levels (b) for which a correlation analysis was performed.

Suppl. Fig. 9 Correlation between microbiome abundance and metabolites (a) 14M faecal microbiome and metabolite (b) 14M microbiome abundance and serum metabolites. (c) Faecal microbiome and faecal metabolites significance levels using Pearson correlation coefficient.

Suppl. Fig. 10 Fragmented α-Syn and expression in ENS from the colon of >12M TG rats. (a) Estimation of fragmentation in α-Syn in from the colon ENS. Two major truncated fragments were found in ENS as shown in α-Syn immunoblot image. (b) Expression of α-Syn in the colon muscular layer.

Suppl. Fig. 11 Accumulation of α-Syn in TG rat colon. (a) Colon tissues were digested with trypsin or Proteinase K for 2 hours (1:5 dilution of the original) and performed with human (TG rats) specific antibodies as described in materials and methods. IHC showed the accumulation of human/rat specific total α-Syn in both 2M and >12M TG rats, whereas in WT (for both the age group) α-Syn was not detected. After digestion aggregated α-Syn was retained in TG rats suggested the accumulation. (b) Detection of aggregated phosphorylated (pS129) α-Syn in the colon tissues in young and old TG rats after trypsin digestion. (c) Proteinase K digestion of the colon to investigate accumulation of aggregated total α-Syn protein in TG rats.

Suppl. Fig. 12 Human α-Syn is truncated in TG rats. (a) The expression of endogenous α-Syn in rat colon and upto 4 different truncated α-Syn can be observed in the colon of the TG rats, but the appearance of these different bands differs between individual and does not seem

to be correlated with age. In some TG rats (5M 1 and 18M 1), the expression of α-Syn is similar than in the WT rats but showing truncated fragments of lower molecular weight. No pathologic phosphorylation (S129, Y125, Y39) could be detected by immunoblotting method. (b). DSP treatment increases α-Syn immunodetection in WB. The detection of the truncated α-Syn band is not dependent on boiling the samples, as similar band is also present in non-boiled samples, but its signal is increased in presence of the crosslinker DSP. (c) Using different α-Syn antibodies covering different epitopes of the sequence were used to detect the fragmentation of α-Syn. N-terminal antibodies did not detect the first truncated fragment present in sample 1 and 2- Detect the second truncated fragment present in sample 2 and 3. Detect an extra truncated fragment in sample 1 with a similar molecular weight the second truncated fragment. C-terminal antibodies do not detect the first and second truncated fragment present in sample 1 and 2, but only upto residue 125. SA3400 (117-131) does not detect these truncated forms. The extra truncated fragment in sample 1 is detected by all c-terminal antibodies. 1st truncated band is observed with α-Syn 211 (121-125), 14H2L1 (117-125), LB509(115-122) and FL140 (61-95), however it was not observed with SA3400 (117-131), EP1466Y (N-term), N19 (N-term) and Ab6176 (11-26). The fact that the 1st truncated band was not observed by SA3400 (117-131), however it was observed by all the other C-term antibodies upto 125, suggest that it might be a N-terminal fragment with the truncation located between the position 125 and 131. Further, the truncated fragment was not observed by any of the three N-term antibodies tested, thus further suggesting that the fragment was also truncated at N-terminal part. The 2nd band was observed with N19 (N-term), Ab6176 (11-26), EP1646Y (N-term), 14H2L1 (117-125) and LB509 (115-122) and not observed with SA3400 (117-131). Thus, detected band was observed by all the N-term antibodies but not by the most distant C-term antibody (SA3400) suggesting that it was truncated in the C-terminal part, probably around the position 120. (d) A cartoon is showing possible aberrant alternative splicing variant sites.

Suppl. Fig. 13 RNA-seq analysis from 3M and >12M old WT and TG colon epithelial tissues. (a) Differentially expressed genes at 3M age in WT and TG rats. Total no of differentially expressed genes (153) based on absolute log fold-change of at least 1 (raw p value P=0.05), whereas at >12M age total no of differentially expressed were 1808 based on absolute log fold-change of at least 1 (raw p value P=0.05). There was no statistical significance based on false discovery rate (FDR) for 3M, however at >12M 264 genes were different. (b) Clustering of >12M WT and TG epithelial (Epi) samples. (c,d), IPA analysis of adaptive (Th1 pathway) and innate (TLR pathway) for the >12M samples. (e) Different inflammatory gene expressions

Suppl. Fig. 14 Reactome transcriptome wide overview analysis of 3M and >12M epithelial RNA-seq samples. The figure shows a genome-wide overview of the results of pathway analysis (a) 3M and (b) >12M. Reactome pathways are arranged in a hierarchy. The center of each of the circular “bursts” is the root of the one top-level pathway. Each step away from the center presents the next level lower in the pathway hierarchy. The colour code denotes over-representation of a particular pathway in the input dataset. Light grey signifies pathways which are not significantly over-presented. At 3M programmed cell death pathway genes were over-presented whereas at >12 M programmed cell death pathways gene were downregulated. Further, at >12M M there were an over-representation of immune system genes and less representation of programmed cell death genes.

Suppl. Fig. 15 Antibiotics treatment strategy and health status of the rats. (a) Antibiotics treatment plan for young rats. (b) Antibiotics treated health based on body weight.

Suppl. Fig. 16 Antibiotics treatment increase TRPV1 and Dnase1 in the colon. (a) Clustering of the samples in the control (WT and TG) and antibiotics treated groups. (b) Genotype analysis in control and antibiotics treated colon samples (epi). Total 165 genes were found to be commonly regulated by antibiotics. More differential gene expression in WT compared with TG rats. (c) Expression of TRPV1, Dnase1 in WT and TG colon.

Suppl. Fig. 17 Metabolites changes in the feces and serum after antibiotics treatment at 3M age. (a) Antibiotics treatment results in a clear separation of feces samples in the (a) PLS-DA analysis (b) and heat map. (c) While the effect on the corresponding serum scores plots and (d) heat map is rather small. (e,f,g) Multivariate VIP scores analysis identified decreased succinate in both TG and WT control samples after antibiotics treatment alongside the ketone body 3-Hydroxybutyrate. Box and whisker plots showed the succinate and 3-Hydroxybutyrate in control and antibiotic treated samples.

Suppl. Fig. 18 Succinate treatment increase the chemokine & inflammatory (MCP1/IL-12) pathway proteins in neuroblastoma cells. (a) SH-SY5Y neuroblastoma cells were treated with different concentrations succinate as mentioned. Supernatants were collected from each condition and subjected to 13-plex cytokine analysis. MCP1 was significantly increased after 100 μM succinate treatment. Paired Student's T-test was applied for statistical analysis. P value significance represents *$p \leq 0.05$. (b) GPR91 or SUCNR1 expression in SH-SY5Y neuroblastoma cells (n=2). (c) Volcano plots for differentially regulated proteins. (d) Pathway analysis for downregulated (e) upregulated expressed proteins (Control vs 100 μM succinate).
